# Supplementary material for: FTIR-derived soil degradation indices and stochastic modelling of organic matter–sediment dynamics in a Mediterranean watershed: A Northern Apennines case study
Source: PLoS One. 2025 Aug 21;20(8):e0330252. doi: 10.1371/journal.pone.0330252 (PMC12370054; doi:10.1371/journal.pone.0330252)
Supplement: S1 Appendix — (PDF) [file pone.0330252.s001.pdf]

Supporting Information

S1 Appendix. Additional information regarding laboratory procedures and methodology.

Laboratory procedure for analysing fluvial sediment samples through FTIR Spectroscopy.

For each sample, we grinded 1 g of material with a mechanical mill (MM20; Retsch, Germany) to reduce particle diameter to ≤ 20 μm, as finer particles improve the accuracy and quality of FTIR spectral data, enhancing the predictive models for soil properties [157,158]. Then, 1 mg of grinded material was mixed with 100 mg KBr and stored for 24 hours in an exicator over dried silica gel to standardize the water content. Subsequently, dried samples were finely grinded using an agate mortar and transformed into pellets using a 9 Mg compactor [50]. The pellets were then analyzed using a Nicolet iS20 Spectrometer (Thermo Scientific), in combination with the OMNIC software v. 9.13.1224, to obtain the absorption spectra of sediment in a range of wavenumbers (WN) between 3900 and 400 cm^{-1} [82]. All spectra were recorded under the same conditions: 16 repetitions of a single spectrum, at a resolution of 2 cm^{-1}, and a data spacing of 0.241 cm^{-1}. Additionally, we performed the background correction before each sample analysis, which accounts for the composition of the air in the measurement chamber [83]. We further processed raw spectra data using the Resolution Pro (Agilent) software v. 5.3.6.1694. Before interpretation, all spectra were smoothed using a "boxcar" function (f = 25) and corrected for baseline.

Methods used for calculating terrain parameters through DTM-based Terrain Analysis.

Slope, *Aspect*, *Profile Curvature*, *Tangential Curvature*, *General Curvature*, and *Total Curvature* were calculated using the 9-parameter 2nd order polynomial method according to Zevenbergen & Thorne [103]. The *Downslope Distance Gradient* was calculated as the ratio of the vertical drop to the horizontal flow path length, using a vertical distance parameter of 10 m, to quantify downslope controls on local drainage [104]. The *SAGA Wetness Index* was calculated as the natural logarithm of the ratio between the specific catchment area and the tangent of the local slope angle [92]. The *Terrain Ruggedness Index* was calculated as the mean difference in elevation between a central pixel and its surrounding cells in the DTM according to Riley et al. [105]. The *Stream Power Index* was calculated as the product between the specific catchment area and the tangent of the slope angle according to Moore et al. [94]. The main channel stems (i.e., Lubiana creek and Arda river) were used to derive the *Channel Network Base Level* (CNBL) from the DTM, which represents the elevation of the channel network that acts as an erosional reference "base" for the surrounding landscape. In other words, lower CNBL values indicate areas where the fluvial system corresponds (or is close) to the erosional base level at the valley bottom, thus

shaping the topographic features of the landscape, while higher CNBL values represent areas far from the reference base. Subsequently, we subtracted the CNBL from the original elevations in order to derive the *Vertical Distance to Channel Network* [102,159,160].

Additional references

157. Le Guillou F, Wetterlind W, Rossel RV, Hicks W, Grundy M, Tuomi S. How does grinding affect the mid-infrared spectra of soil and their multivariate calibrations to texture and organic carbon? Soil Res. 2015;53(8):913-921. doi: 10.1071/SR15019.
158. Deiss L, Culman SW, Demyan MS. Grinding and spectra replication often improves mid-DRIFTS predictions of soil properties. Soil Sci Soc Am J. 2020;84(3):914-929. doi: 10.1002/saj2.20021.
159. Adeniyi OD, Brenning A, Maerker M. Spatial prediction of soil organic carbon: Combining machine learning with residual kriging in an agricultural lowland area (Lombardy region, Italy). Geoderma. 2024;448:116953. doi: 10.1016/j.geoderma.2024.116953.
160. Vogel S, Maerker M. Reconstruction of the pre-eruption AD 79 paleo-topography and paleo-environment of the Sarno River Basin (Italy) using stratigraphical core drillings and data mining technologies. Geomorphology. 2010;115(1-2):67-77. doi: 10.1016/j.geomorph.2009.09.031.
