# Supplementary material for: FTIR-derived soil degradation indices and stochastic modelling of organic matter–sediment dynamics in a Mediterranean watershed: A Northern Apennines case study
Source: PLoS One. 2025 Aug 21;20(8):e0330252. doi: 10.1371/journal.pone.0330252 (PMC12370054; doi:10.1371/journal.pone.0330252)
Supplement: S2 Appendix — (PDF) [file pone.0330252.s002.pdf]

Supporting Information

S2 Appendix. Basics statistics for the raster variables selected as feature variables.

The DTM provided the *Elevation*, ranging from 290 to 1352 m, with a mean of 757 m and a standard deviation (STD) of 220 m. *Flow Accumulation* ranges from 25 to 8.84 x 10^7 m^2, with a mean of 4.56 x 10^4 m^2 and a STD of 1.22 x 10^6 m^2. *Slope* ranges from 0.03 to 58.93°, with a mean of 15.06° and a STD of 7.90°. *Aspect* ranges from 0 to 360°, with a mean of 167° and a STD of 112°. *Profile Curvature* ranges from -0.27 to 0.07 m^{-1}, with both mean and STD of 0. *Tangential Curvature* ranges from -0.33 to 0.11 m^{-1}, with both mean and STD of 0. *General Curvature* ranges from -0.90 to 0.68 m^{-1}, with a mean of 0 and a STD of 0.01 m^{-1}. *Total Curvature* ranges from 0 to 0.13 m^{-1}, with both mean and STD of 0. *Downslope Distance Gradient* ranges from 4.95 to 2374.43 m, with a mean of 60.44 m and a STD of 105.15 m. *SAGA Wetness Index* ranges from 2.16 to 21.75, with a mean of 6.65 and a STD of 2.02. *Terrain Ruggedness Index* ranges from 0 to 2.67 m, with a mean of 0.36 m and a STD of 0.21 m. *Stream Power Index* ranges from 0 to 9.08 x 10^6 m, with a mean of 636.35 m and a STD of 2.92 x 10^4 m. *Channel Network Base Level* ranges from 289.64 to 1263.99 m, with a mean of 553.87 m and a STD of 192.50 m. *Vertical Distance to Channel Network* ranges from -1.86 to 745.81 m, with a mean of 203.24 m and a STD of 136.27 m.
SOC stock ranges from 7.05 to 151.04 Mg ha^{-1}, with a mean of 78.60 Mg ha^{-1} and a STD of 13.08 Mg ha^{-1}. *SOC %* ranges from 1.23 to 4.25%, with a mean of 2.05% and a STD of 0.34%. *Clay %* ranges from 7.30 to 46.60%, with a mean of 28.64% and a STD of 5.39%. *Silt %* ranges from 25.31 to 57.33%, with a mean of 43.10% and a STD of 3.12%. *Sand %* ranges from 10.75 to 65.63%, with a mean of 28.26% and a STD of 7.58%. *Skeleton %* ranges from 0.03 to 30.70%, with a mean of 20.36% and a STD of 7.86%. *pH* ranges from 5.47 to 7.90, with a mean of 7.29 and a STD of 0.61.
