# Supplementary material for: FTIR-derived soil degradation indices and stochastic modelling of organic matter–sediment dynamics in a Mediterranean watershed: A Northern Apennines case study
Source: PLoS One. 2025 Aug 21;20(8):e0330252. doi: 10.1371/journal.pone.0330252 (PMC12370054; doi:10.1371/journal.pone.0330252)
Supplement: S1 Fig — (PDF) [file pone.0330252.s003.pdf]

Supporting Information

S1 Figures - Permutation-based variable importance plots for all the experiments.

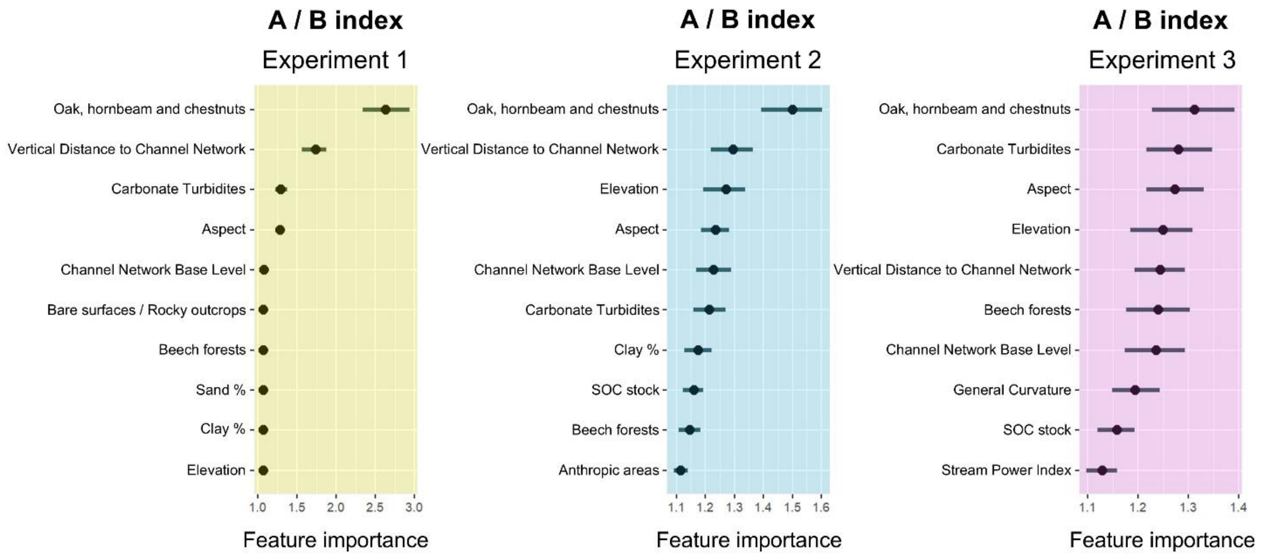

S1.A Figure. Feature importance plots for the A/B index.

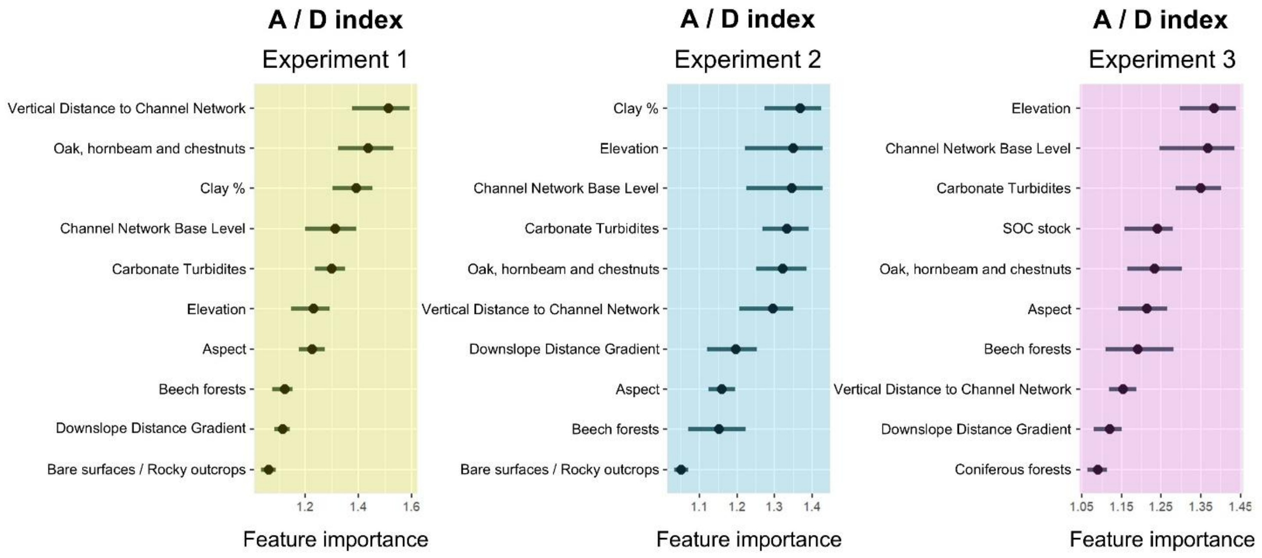

S1.B Figure. Feature importance plots for the A/D index.

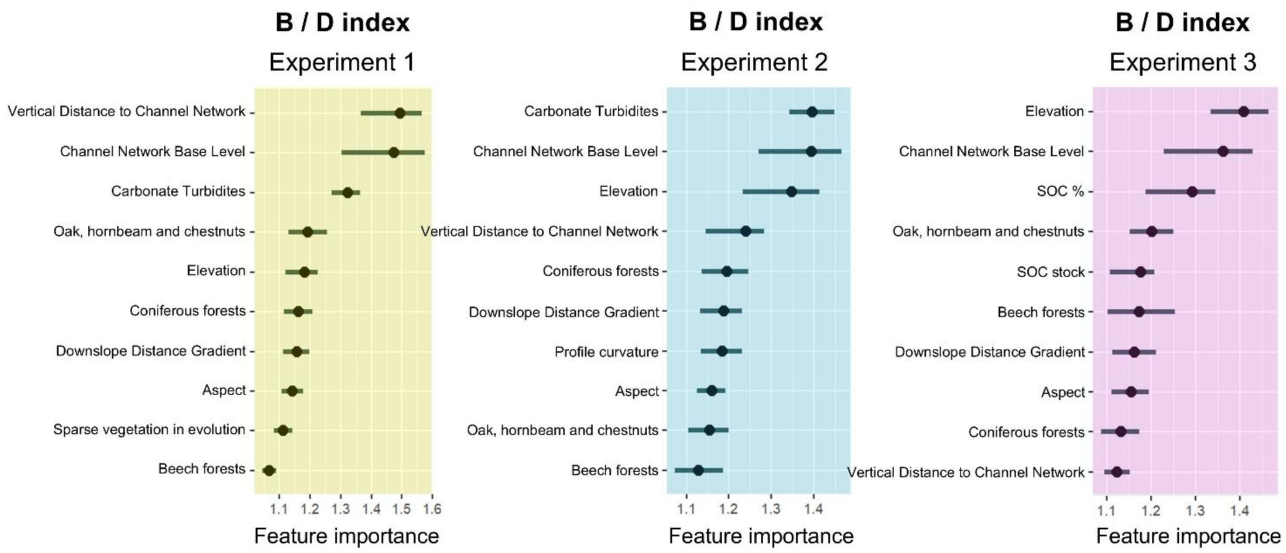

S1.C Figure. Feature importance plots for the B/D index.

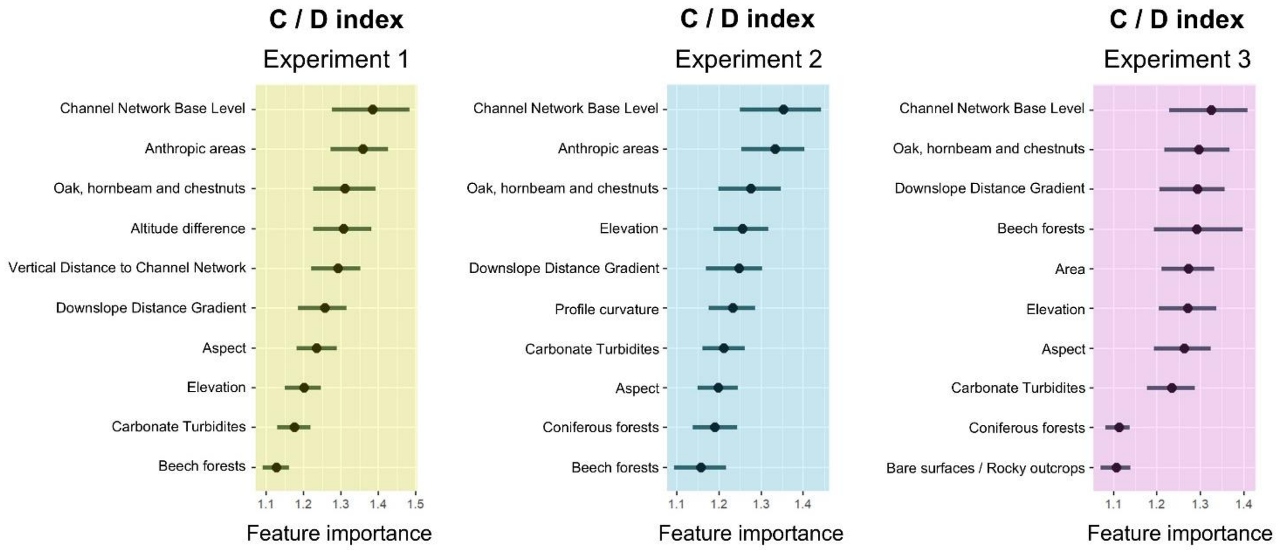

S1.D Figure. Feature importance plots for the C/D index.
