# Supplementary material for: FTIR-derived soil degradation indices and stochastic modelling of organic matter–sediment dynamics in a Mediterranean watershed: A Northern Apennines case study
Source: PLoS One. 2025 Aug 21;20(8):e0330252. doi: 10.1371/journal.pone.0330252 (PMC12370054; doi:10.1371/journal.pone.0330252)
Supplement: S2 Fig — (PDF) [file pone.0330252.s004.pdf]

Supporting Information

S2 Figure - Accumulated Local Effect (ALE) plots for all the experiments.

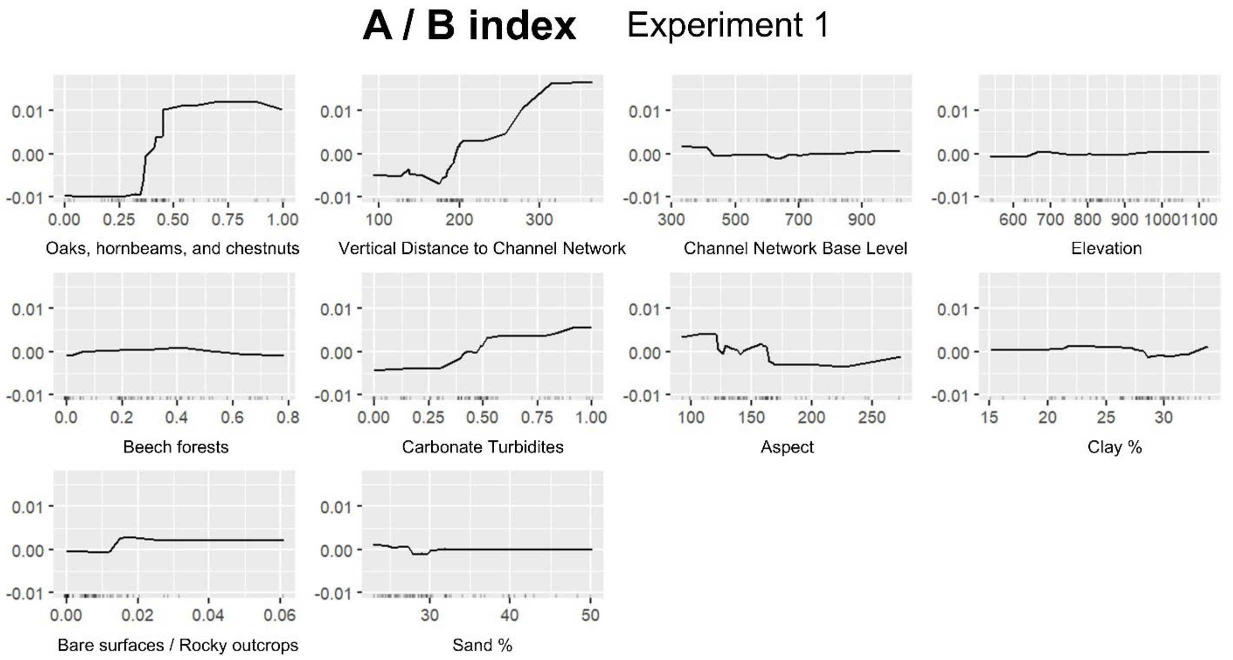

S2.A Figure. Accumulated Local Effect (ALE) plots for the A/B index, Experiment 1.

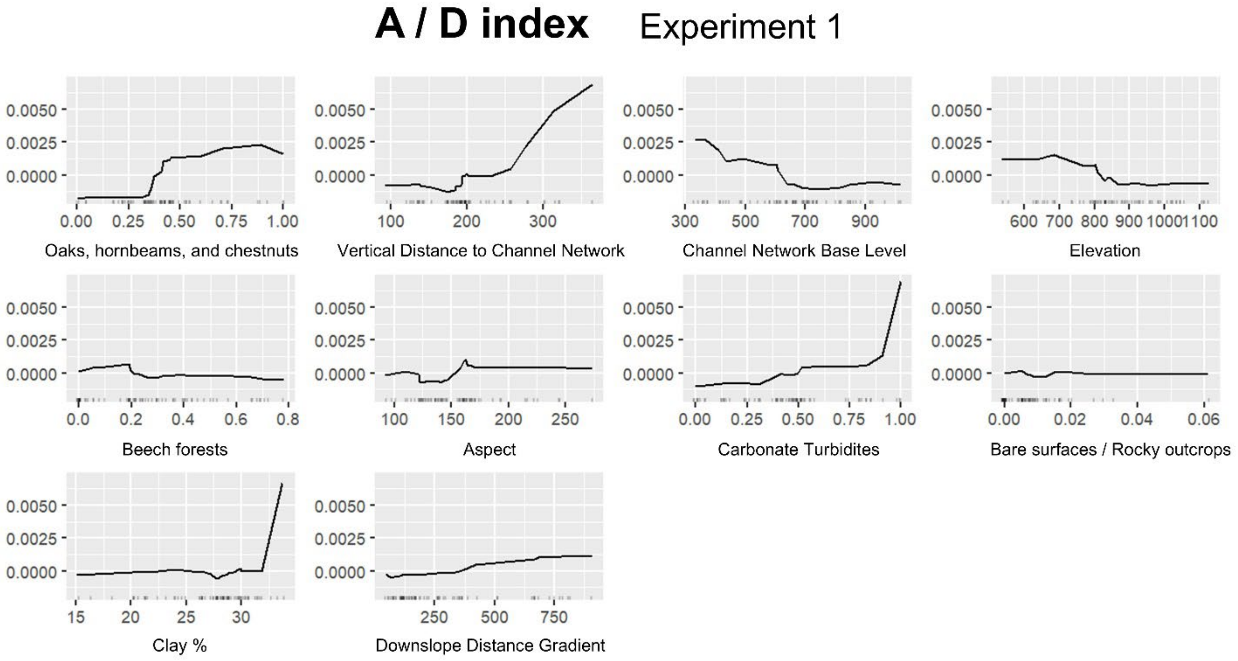

S2.B Figure. Accumulated Local Effect (ALE) plots for the A/D index, Experiment 1.

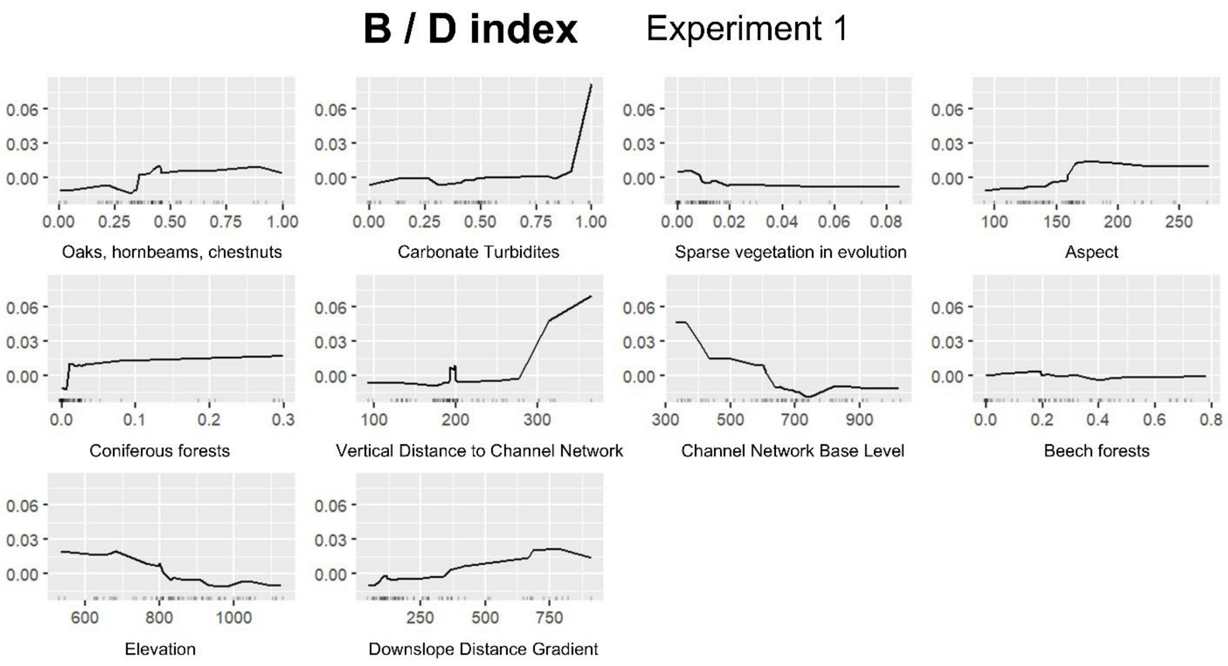

S2.C Figure. Accumulated Local Effect (ALE) plots for the B/D index, Experiment 1.

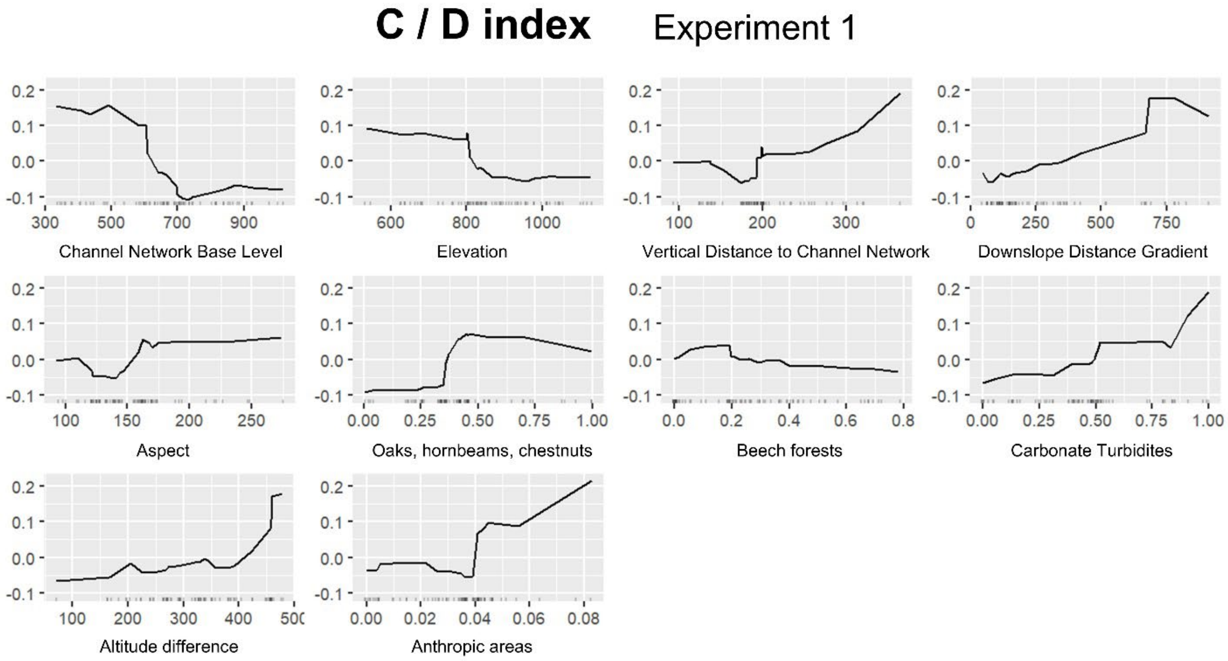

S2.D Figure. Accumulated Local Effect (ALE) plots for the C/D index, Experiment 1.

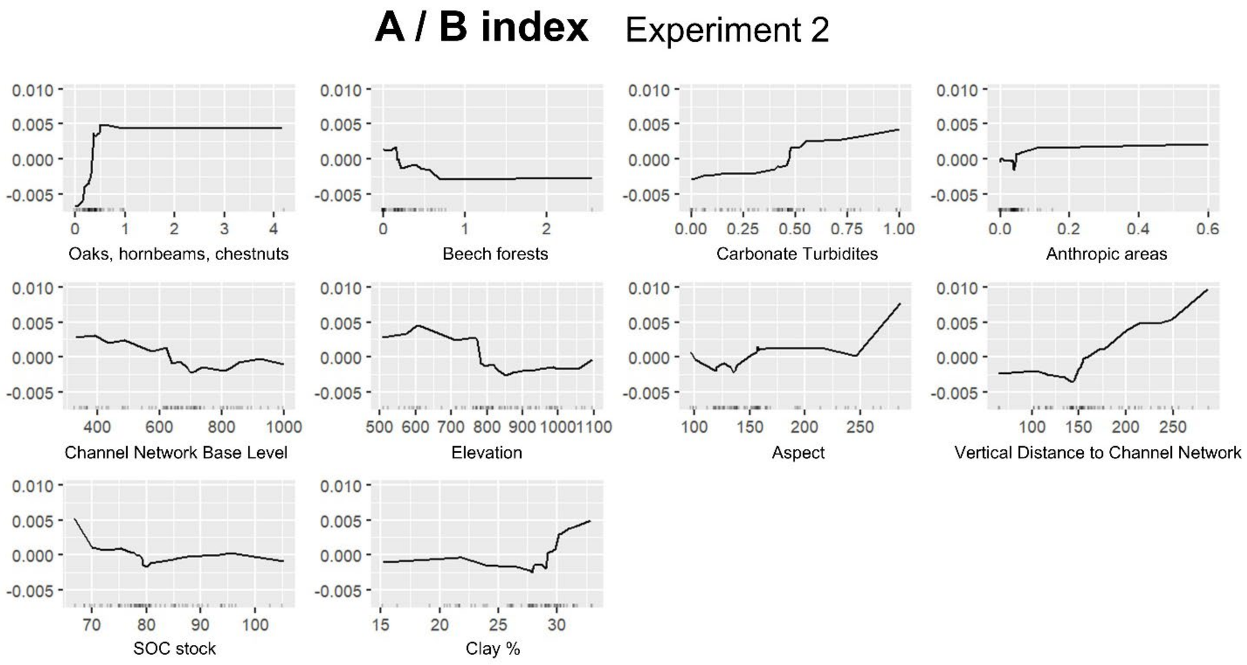

S2.E Figure. Accumulated Local Effect (ALE) plots for the A/B index, Experiment 2.

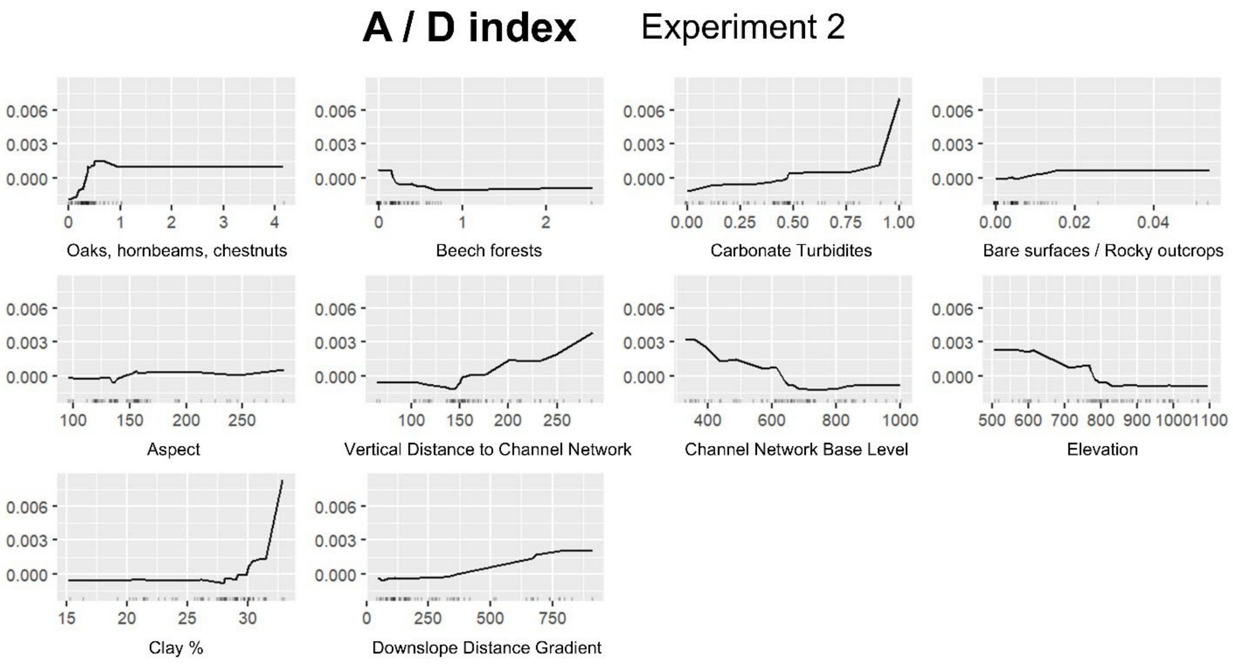

S2.F Figure. Accumulated Local Effect (ALE) plots for the A/D index, Experiment 2.

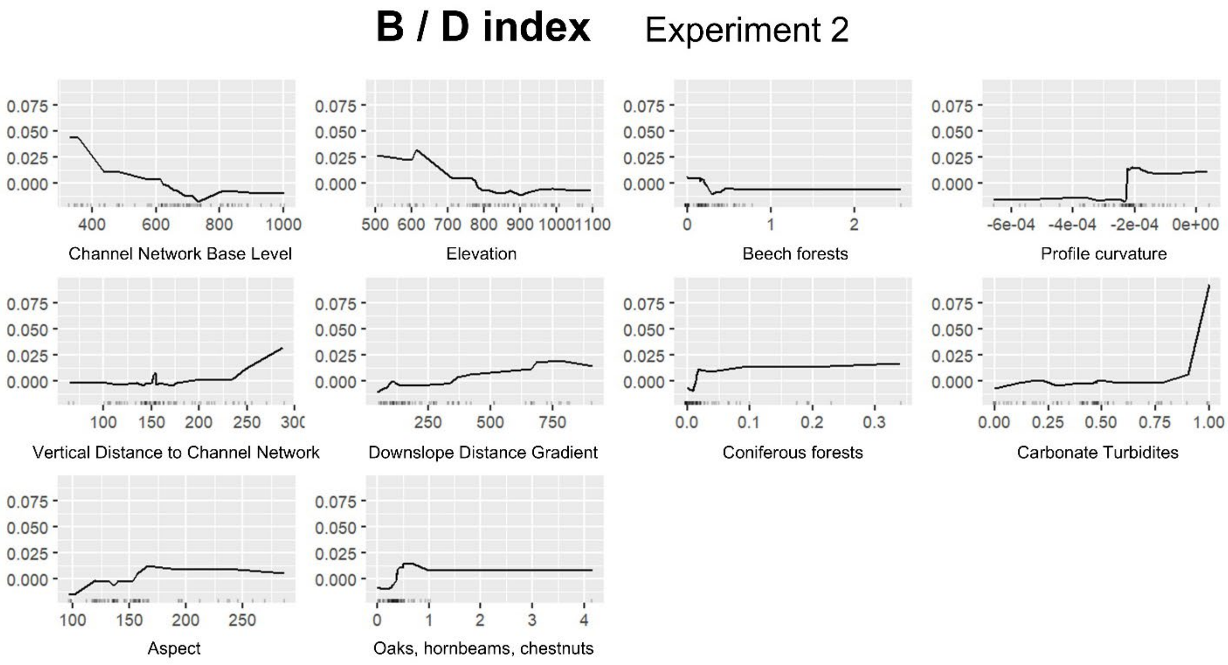

S2.G Figure. Accumulated Local Effect (ALE) plots for the B/D index, Experiment 2.

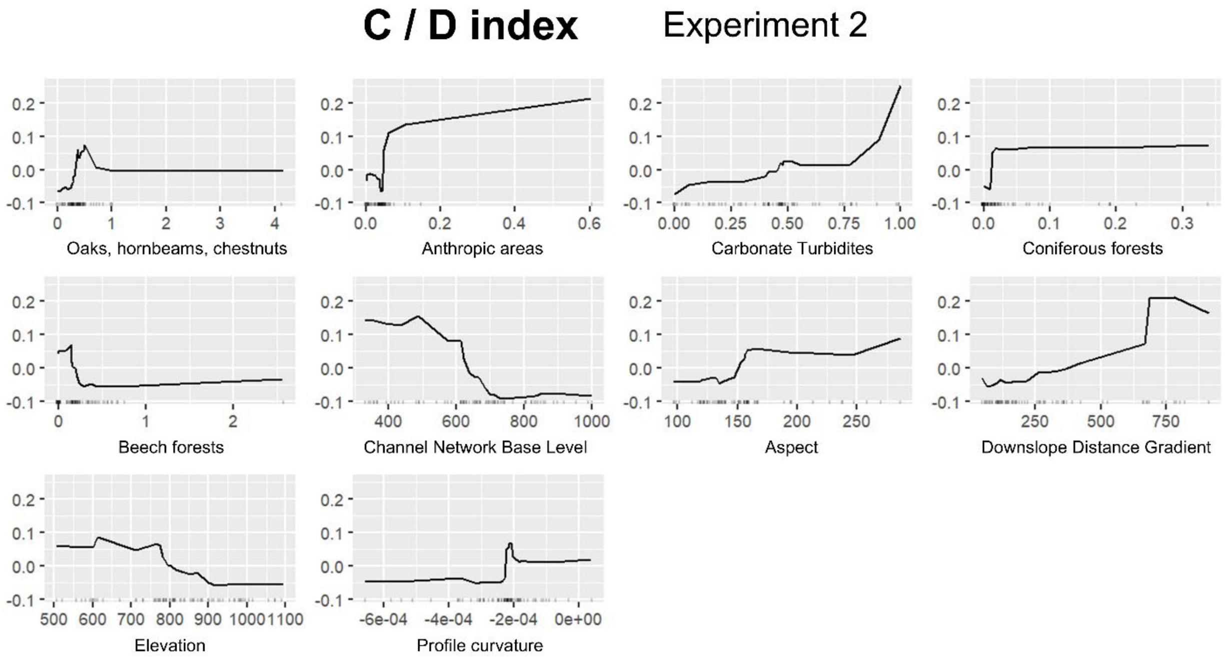

S2.H Figure. Accumulated Local Effect (ALE) plots for the C/D index, Experiment 2.

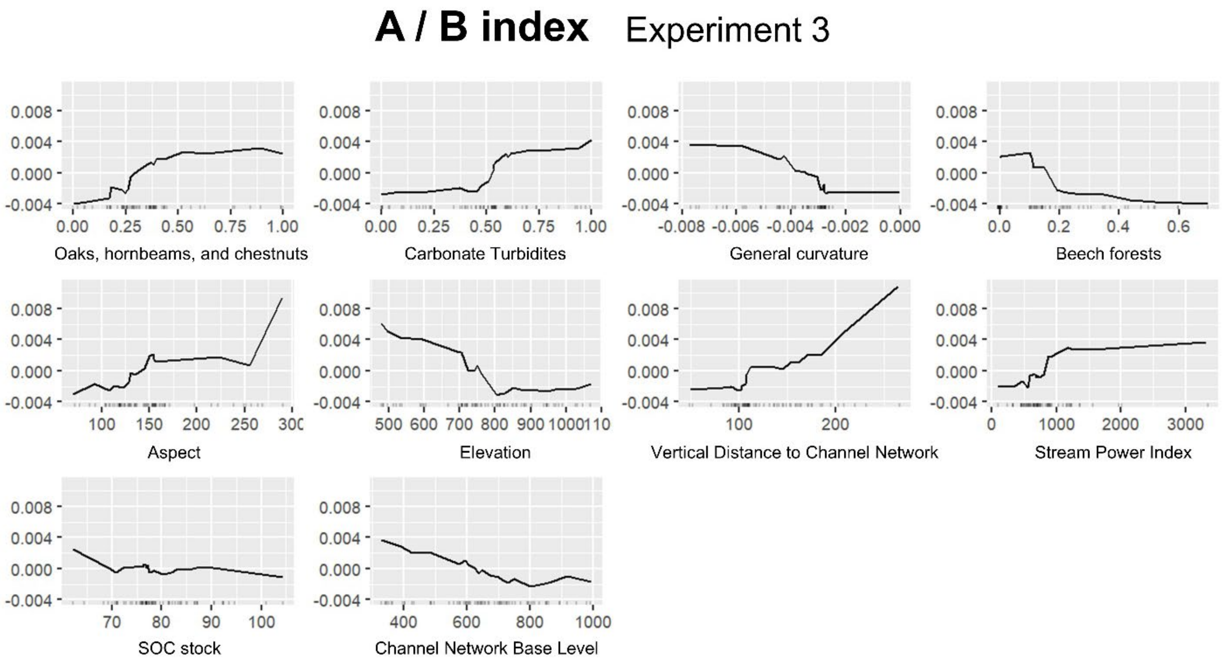

S2.I Figure. Accumulated Local Effect (ALE) plots for the A/B index, Experiment 3.

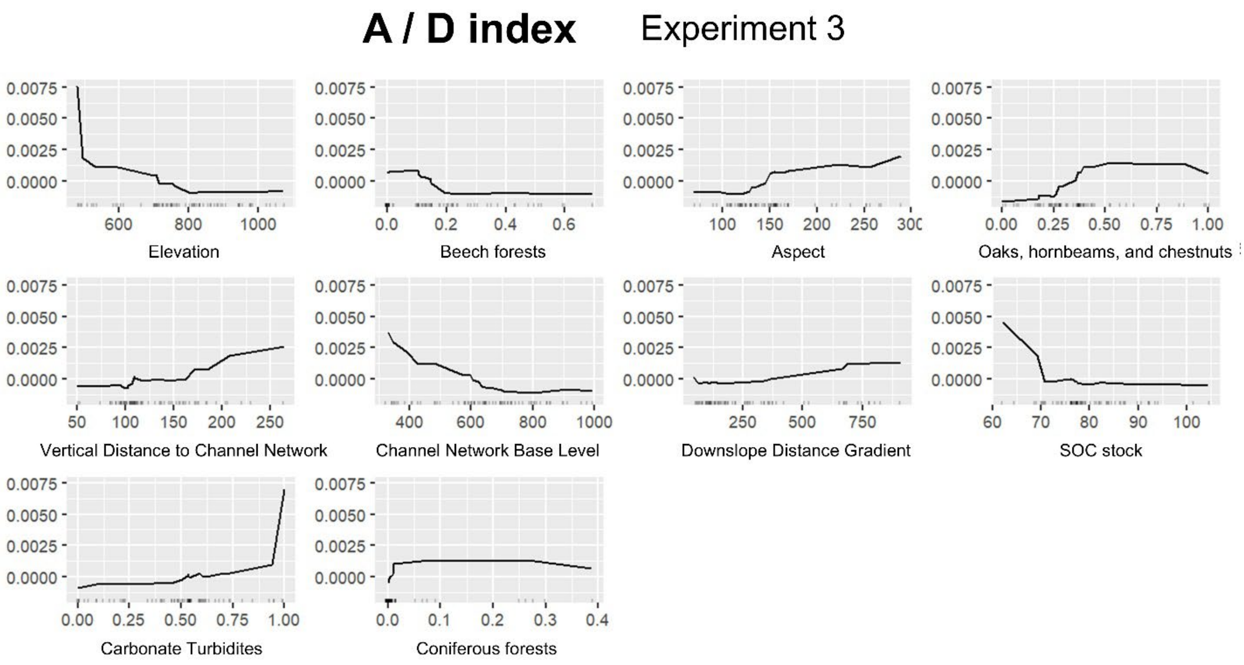

S2.J Figure. Accumulated Local Effect (ALE) plots for the A/D index, Experiment 3.

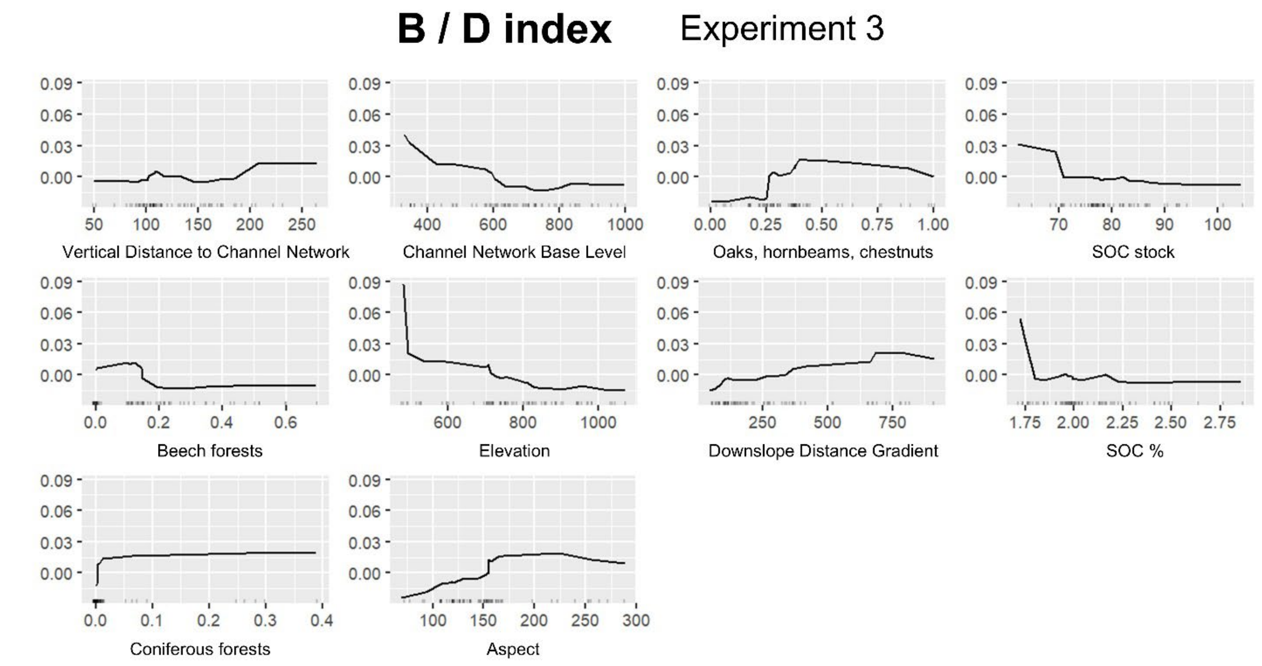

S2.K Figure. Accumulated Local Effect (ALE) plots for the B/D index, Experiment 3.

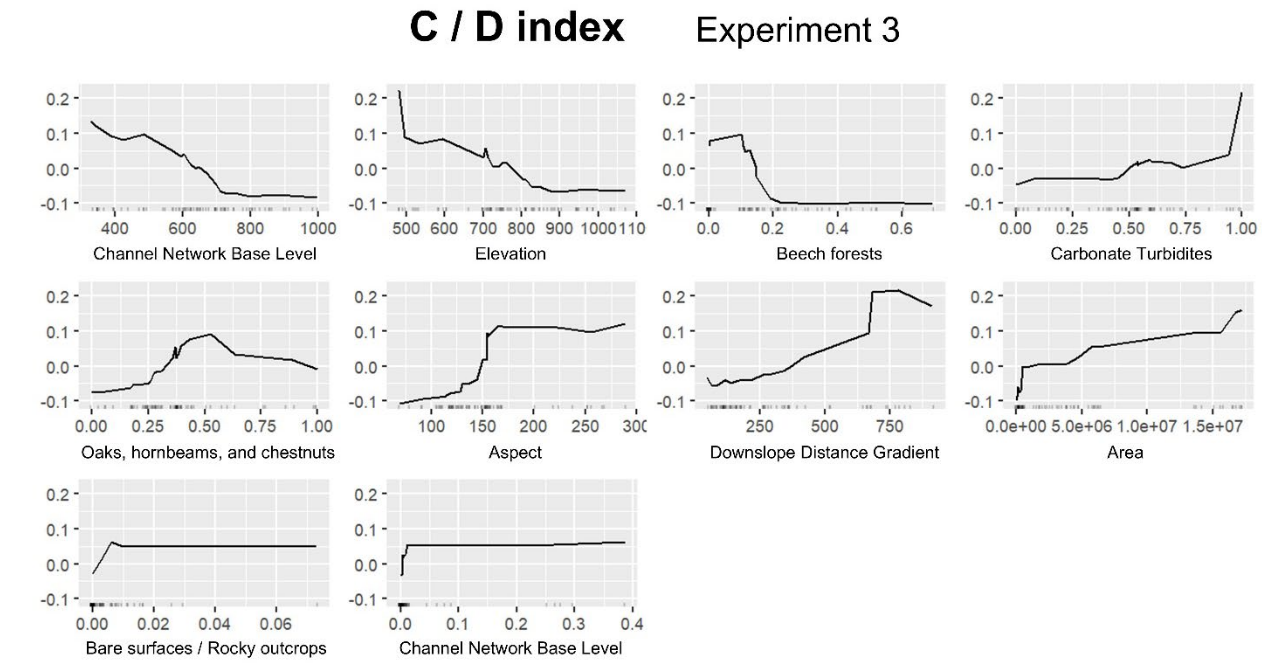

S2.L Figure. Accumulated Local Effect (ALE) plots for the C/D index, Experiment 3.
