# Supplementary material for: FTIR-derived soil degradation indices and stochastic modelling of organic matter–sediment dynamics in a Mediterranean watershed: A Northern Apennines case study
Source: PLoS One. 2025 Aug 21;20(8):e0330252. doi: 10.1371/journal.pone.0330252 (PMC12370054; doi:10.1371/journal.pone.0330252)
Supplement: Attachment — Submitted filename: PONE-D-25-04924 Response_to_Editor.docx [file pone.0330252.s005.docx]

Dear Editor,

We would like to express our gratitude for your valuable comments and suggestions, which have significantly contributed to improve the manuscript. We have tracked the changes throughout the manuscript and provided a clean copy without tracked changes. We have addressed your requests and comments throughout the manuscript. Please find below our responses. The specific references to the modified lines are referred to the “clean copy”.

***Ed_1: The manuscript, while addressing an interesting and relevant topic, does not meet PLOS ONE's publication criteria due to significant structural and presentation issues. The overly long title, misaligned with the abstract, creates a poor first impression and is likely to deter professional reviewers, making it difficult to proceed with the peer review process. The abstract contains excessive technical details and fails to emphasise the study's practical significance, while the keywords merely repeat terms from the title, reducing their effectiveness in search engines. Key sections, such as "Materials and Methods" and "Results," lack clarity and fail to link methods to findings effectively, while the "Discussion" does not critically interpret the results or situate them within the broader literature. Additionally, the "Conclusion" is overly general and does not provide a concise summary of the study’s contributions. The manuscript also deviates from PLOS ONE’s citation formatting guidelines, indicating a lack of compliance with submission standards. These issues collectively make the manuscript unsuitable for publication in its current form, and I encourage the authors to address the detailed feedback provided in the review before considering resubmission. Detailed comments on these issues are provided in the context of each section discussed below. To increase the chances of engaging both reviewers and readers, substantial editorial and substantive revisions are necessary. I kindly request that you consider these suggestions during the revision process and warmly invite you to resubmit your manuscript to our journal.***

Corresponding author (CA): We really appreciated your attention, interest and efforts during the review process of our article. Thank you so much! We closely followed yours comments and suggestions for every section of the manuscript. We have deeply changed and modified the whole manuscript to accomplish a new version. We have solved structural and presentation issues arisen from your revision. Thus, we strongly believe this new version, resulted from your revision, will align with standards for publication in PlosONE. Please find below our point-by-point responses. Please consider that all the sections of the manuscript have been changed.

***Ed_2: The title of the manuscript, while covering all key aspects of the study, is overly long, which negatively impacts its readability and compliance with PLOS ONE guidelines. The excessive detail in the title hinders the swift understanding of its content, diminishes its appeal, and may reduce the manuscript's potential for citations. Much of the information currently included in the title could be effectively moved to the abstract, allowing for the creation of a more concise and accessible title. Shortening the title is essential to improve its clarity and alignment with the journal's requirements. A key element of these changes is improving the title, which is currently too long, detailed, and misaligned with the content of the abstract, negatively impacting its reception and potentially deterring reviewers and readers. I propose a more concise and precise title that better reflects the essence of the study: "Integration of FTIR-based soil organic matter indices with stochastic modelling for analyzing erosion and sediment dynamics in a Mediterranean watershed". This title retains the key information, emphasises the interdisciplinary approach, and is more appealing to a broader audience.***

CA: Thank you for this comment. The previous version of the title was “*FTIR-based indices of soil organic matter and stochastic modelling reveal insights into spatial patterns of erosion processes, sediment dynamics, and organic matter connectivity at watershed scale: An interdisciplinary study*”. It covered 29 words and 224 characters (with spaces included). We agree that the title was excessively detailed. We have changed and shortened the title accordingly: “***Integration of FTIR-based soil degradation indices with stochastic modelling to assess spatial patterns of organic matter-sediment dynamics in a Mediterranean watershed***”. We believe this new version of the title better reflects the content of the study and aligns with the abstract. Now the title has 21 words and 168 characters (with spaces included). Please consider that differently than your proposed title, we have only added the reference to organic matter dynamics, thereby changing “soil organic matter indices” with “*soil degradation indices*” to avoid redundant words. We strongly believe that the concept of organic matter dynamics is one of the key and novel aspects of our study. Moreover, we prefer to speak about “*spatial patterns*” of these dynamics in the title, as clearly discussed in the abstract. Our proposed new title is longer than just 1 word than yours.

***Ed_3: The keywords in the manuscript repeat terms from the title, which is inconsistent with PLOS ONE guidelines and limits their effectiveness in scientific search engines. The lack of diversity and additional context results in key aspects of the study, such as soil erosion analysis, the specificity of the Mediterranean watershed, or the application of spatial modelling, being insufficiently represented. Moreover, overly generic terms, such as "Interdisciplinary Approach," fail to effectively reach the intended audience. To enhance the visibility and impact of the work, it is necessary to replace repetitive and generic keywords with more precise ones that better reflect the interdisciplinary nature and findings of the study. I suggest the following: "soil erosion," "Mediterranean watershed," "spectroscopic analysis," "geomorphological modelling," "sediment connectivity," "organic matter indices." This set avoids redundancy, better reflects the interdisciplinary nature of the study, and more effectively highlights its key aspects.***

CA: Thank you for this comment and for proposing new keywords. We have replaced the keywords closely following your comments and suggestions: *Erosion susceptibility*; *Organic matter indices*; *Spectroscopic analysis*; *Mediterranean watershed*; *Geomorphological modelling*; *Landscape connectivity*. We believe that these new keywords now reflect key aspects of the study, such as soil erosion assessment, the use of organic matter indices derived from spectroscopic analysis, the specificity of Mediterranean environments, the application of geomorphological spatial modelling, and landscape connectivity. We avoid generic terms such as “Interdisciplinary approach”, as the interdisciplinary nature of this study is evident from the title and keywords themselves.

***Ed_4: The abstract effectively presents key aspects of the study, including its objectives, methodology, and results. However, its length and the inclusion of excessive technical details reduce its readability and accessibility for a broader audience. Technical terminology, such as "Leave-One-Out Cross Validation" or "Vertical Distance to Channel Network," is unnecessary in the abstract and should be moved to the methodology section. Additionally, the abstract does not sufficiently highlight the practical significance of the study’s findings for soil conservation or environmental management in Mediterranean regions, which could increase its relevance for readers from various fields. In its current form, the abstract lacks a clear summary that succinctly emphasises the importance of the results, which limits its ability to fully meet the interdisciplinary nature required by PLOS ONE. Focusing on the key conclusions and their significance while reducing unnecessary technical details would significantly improve its readability and effectiveness.***

CA: Thank you for this comment. We have shortened the abstract (261 vs. 274 words) and removed technical details and too specific terms in order to improve the readability and accessibility for a broader audience. Thus, we believe this abstract is more fluent and accessible to readers from all disciplines. Now the abstract efficiently summarizes the 1) **general** **aim** “*In this study we explored the relationships between Soil Organic Matter (SOM) properties, serving as potential indicators of soil degradation and erosion, and environmental, geomorphic, and hydrological characteristics in an agricultural-forested Mediterranean watershed*”, 2) **methodology** “*SOM composition of fluvial sediments sampled across the watershed was analysed using FTIR spectroscopy to calculate FTIR-based proxies for the relative hydrophobicity of SOM, Cation Exchange Capacity (CEC), and organic matter-cation associations. To investigate geospatial relationships between SOM composition influencing erosion susceptibility and the factors driving its variability at the watershed scale, such as terrain characteristics, soil properties, lithological, and LULC data, we used a Random Forest modelling approach*”, 3) **findings highlighting the key conclusions and the significance of the study** “*Our findings indicate that the size and configuration of the contributing areas associated with the sampling points played a crucial role in interpreting the relationships between SOM composition and environmental factors. Oak, hornbeam, and chestnut forests influence hydrophobic organic matter accumulation, making soils more prone to water erosion, where clay content potentially intensifies erosion susceptibility under particular climatic conditions. Moreover, SOM chemical components were spatially linked to sediment dynamics and organic matter connectivity across the watershed, with topographic features such as elevation and channel network base level being key factors. Also, CEC was found to be a potential indicator of soil erosion in geomorphologically active areas. Lastly, carbonate-rich soils appeared to positively influence organic matter-cation associations, potentially enhancing aggregate stability and reducing erosion susceptibility*”, and 4) **scientific and practical significance of this study** “*This study provides significant new insights into the complex relationships between SOM composition, environmental predictors, and soil erosion in Mediterranean watersheds, supporting novel research hypotheses and perspectives from both a scientific and applicative point of view*”.

***Ed_5: The "1. Introduction" section introduces the study topic, emphasising the role of soil organic matter (SOM) in maintaining soil quality and the importance of an interdisciplinary approach to analysing erosion and sediment dynamics. Despite the wealth of information provided, the section is overly detailed, which dilutes its main message and risks discouraging readers. The excessive references and inclusion of technical details, such as SOM mechanisms, should be reduced or moved to the relevant methodological sections. Furthermore, the section lacks a clear focus on the study’s objectives and innovative aspects, as well as an explanation of the rationale for selecting the study location in the context of its environmental specificity. Improving the structure, reducing superfluous details, and emphasising the significance of the study would enhance the clarity and effectiveness of this section..***

CA: Thank you for this comment. We have deeply modified the Introduction section according to your comments. We have reduced the length of the section, removing overly detailed and verbose information, such as specific SOM mechanisms, as well as unnecessary references. Moreover, we have stressed further the focus of our study, highlighting the objectives and innovative aspects. Moreover, we have added references to Mediterranean environments throughout the whole Introduction, emphasizing the rationale for selecting a Mediterranean watershed and, in particular, the environmental specificity of our study area. From a schematic point of view, now the Introduction section is composed of: 1) initial paragraph introducing **soil as a vital resource for social-ecological systems** and its **vulnerability to degradation triggered by geomorphic activity**, especially in Mediterranean environments; 2) a paragraph introducing **Soil Organic Matter (SOM) as a key factor for the soil-environment interface**, along with **SOM-related properties that can be used as indicators of soil degradation susceptibility**, particularly to **water erosion**; 3) a paragraph which illustrates **SOM properties related to chemical composition** such as **hydrophobicity**, **cation exchange capacity** and **organic matter-cation associations**, which will be the topics of the study, briefly introducing their role as potential indicators, with a focus on the Mediterranean environments (avoiding technical details); 4) a paragraph introducing the **watershed-scale interplay between SOM chemical characteristics, environmental features, and landscape connectivity**, briefly illustrating recently published works focusing on **organic matter and sediment dynamics in relation to environmental factors spatial patterns.** This paragraph also stresses **the need for more detailed studies focusing on these complex relationships**, which actually remain relatively underexplored; 5) a paragraph explaining the **aims of the study and the selection of the study area based on its environmental specificity**; 6) a paragraph introducing **Fourier-transform infrared (FTIR) spectroscopy as the main experimental technique used for investigating SOM composition** (removing most of the verbose and technical information included in the old version of the Introduction); 7) a final paragraph briefly summarizing the **whole methodology used for accomplish the study’s aims along with the novel and innovative aspects of the study**.

***Ed_6: The "2. Study area" section provides detailed information about the research location, covering geographical, climatic, geomorphological, and lithological aspects. However, its structure and clarity are insufficient. The inclusion of overly detailed data, such as precise descriptions of lithology and detailed climate statistics, is unnecessary at this stage and detracts from the focus on the study's key aspects. There is a lack of a clear connection between the characteristics of the area and the study's objectives, making it difficult for readers to understand why this location was chosen and how its environmental features influence the studied processes of erosion and sediment dynamics. Incorporating a more thorough analysis of the relationship between land use and erosion could significantly enhance the value of this section. Currently, the section requires simplification, reorganisation, and a stronger emphasis on the link between the environment and the study’s objectives to better serve its purpose within the manuscript.***

CA: Thank you for this comment. The Study area section has been deeply changed according to your suggestion. We have strongly improved its structure and clarity, in relation to the study’s objectives. The section has been simplified and reorganized. All the previously included information has been summarized (removing technical details and climatic statistics) and linked to the study’s objectives. In particular, we have further stressed the link between the characteristics of the study area and the objectives of the study (please see lines 136-139 of the clean copy). However, we would like to point out that geological/lithological information included are not such precise and detailed, as the description provided only accounts for a lithological grouping based on similar characteristics. The description of the lithology of the study area would be considerably more in-depth and detailed, but we have limited this part introducing only essential information at this level (see lines 140-146 of the clean copy). Anyway, please consider that lithological, vegetation, land cover, and soil characteristics have been linked more effectively to the studied processes and sediment dynamics as requested by you, thereby avoiding self-serving information (check lines 147-165 of the clean copy).

***Ed_7: The "3. Materials and methods" section is detailed, but its clarity and organisation leave much to be desired. The inclusion of overly technical descriptions, such as specific parameters like "Vertical Distance to Channel Network" or "SAGA Wetness Index," is overly verbose and at times overwhelming, making it difficult to understand the essence of the methods used. There is no clear division into key stages of the research, nor a clear connection between the methods and the study's objectives, which makes it challenging for readers to identify the most important information. Additionally, the section lacks a critical analysis of the techniques employed, such as the potential limitations of the Random Forest model or errors associated with GIS analyses, which undermines its value. Some details, such as laboratory equipment settings, could be moved to the appendices to improve readability. In its current form, the section is overloaded with technical details and requires reorganisation and a critical examination of the methods to better fulfil its role.***

CA: Thank you for this comment. The Materials and Methods section has been almost completely modified. First of all, **we introduced an initial paragraph for introducing the step-by-step methodology before listing all the sub-sections**: “*In order to accomplish the objectives of this study, we adopted an interdisciplinary methodological workflow as shown in Figure 2: 1) to assess and visualize geospatial data and prepare the sampling design, we delineated the watershed boundaries and the channel network through GIS-based hydrological modelling from a Digital Terrain Model (DTM); 2) we conducted a sampling campaign to collect fluvial sediments across the watershed from the main channels and their tributaries; 3) samples collected were analysed through Fourier Transform Infrared (FTIR) spectroscopy to characterize SOM composition and calculate four different FTIR proxies (i.e., indices) as indicators of soil erosion susceptibility. A dataset of ‘target variables’ was then compiled using values of the calculated indices; 4) several environmental variables including terrain features, soil properties, lithological and LULC datasets were collected in a GIS environment; 5) for each sampling point, we extracted data of the environmental variables from the respective upstream contributing areas through three experimental designs, accounting for different hydrological and geomorphological settings. Then, three datasets of ‘feature variables’ were compiled using values extracted from the variables used; 6) the datasets were finally analysed through a RF machine learning approach to assess the non-linear and geospatial relationships between target and feature variables*” (see lines 167-180 of the clean copy).

Accordingly, **Figure 2 has been changed in order to better emphasize the distinction between different steps of the methodology**. This implies a deep reorganization of the methodology section. In particular, we have aggregated and grouped some methodological parts in a more coherent and logical way based on the workflow adopted (see Figure 2). Hence, this new methodological workflow better outlines the division into key stages of the research, emphasizing the connection between the methods and the study's objectives.

Sampling of fluvial sediments (Sub-section 3.2) has been moved to the beginning of the workflow, after watershed and channel network delineation (useful for sampling design preparation; Sub-section 3.1), followed by FTIR analysis of the samples (Sub-section 3.3). Regarding FTIR sub-section, we have moved information on laboratory procedure, equipment, specific settings, tools and Softwares to Supporting Information (see S1 Appendix; as requested).

Moreover, we have introduced an aggregated sub-section concerning the preparation of environmental variables datasets (Sub-section 3.4), which encompasses information previously distributed into different sub-sections (cfr. The Tracked Changes copy). For instance, the sub-section 3.4 now includes both terrain analysis and acquisition of pre-existing datasets concerning soil properties, lithology, and land use/land cover. This reorganization better reflects how these datasets have been used within the methodological workflow. Furthermore, information on the methods used for calculating terrain parameters through DTM-based Terrain Analysis, including verbose and overwhelming technical description of specific parameters like “Vertical Distance to Channel Network" or "SAGA Wetness Index”, has been moved to Supporting Information as well (see S2 Appendix). However, we keep information included in Table 2, which schematically illustrates the geomorphological significance associated to each terrain variable, as we consider this information essential for clarity and understanding of the findings.

Then, we have illustrated variable extraction based on different experimental designs (Sub-section 3.5), reorganizing information in order to improve the clarity.

Finally, we have illustrated the application of the Random Forest machine learning model (Sub-section 3.6) highlighting the rationale of selecting specific methods and packages based on study’s objectives.

In general, we have improved the Material and methods section with some details concerning the critical analysis of the techniques employed, as well as the potential limitations of the Random Forest model or errors associated with GIS analyses. For instance, we have stated and explained why our sampling approach ensures a comprehensive coverage of the watershed (see lines 195-199 of the clean copy). Moreover, we have explained that our sampling design was aimed at capturing a certain “*uniformity in deposition conditions among the samples as it is critical for maintaining high organic content and preserving biochemical properties. When sediments are deposited under similar hydrodynamic conditions, they generally exhibit more consistent physical and biochemical properties, which is crucial for comparative analysis*” (see lines 206-2010 of the clean copy). Furthermore, when selecting location for sampling, we have stated that “*particular attention was paid to select locations that remain unaffected by water rise from the main stream during flood events, thus maintaining the integrity of the samples*” (see lines 213-215). In the sub-section 3.4, we have clearly stated the resolution of every raster dataset we used and the methods used for reseampling. In particular, we have added a part explaining that “*All the datasets are provided along with the related pixel-based cartographic accuracy (i.e., low, medium, or high accuracy), defined as the standard deviation of the estimated value in each pixel (i.e., the lower the standard deviation, the more accurate the estimate). For the study area, all the datasets have, on average, a medium accuracy. Further details concerning the full methodology (i.e., spatial prediction, validation, regionalization, and accuracy evaluation) can be found in Ungaro et al. [106-109]. Afterwards, all the datasets were resampled at 5 m resolution using the Nearest Neighbour method. Due to the nature of the data, the initial resolution and processing methods of these datasets may potentially introduce errors and uncertainties related to data representation and spatialization. Nevertheless, resampling and alignment with outputs derived from the DTM ensures consistency in spatial resolution, which is essential for accurate and homogeneous pixel value sampling across all datasets and for reliable statistical analysis*” (see lines 278-287 of the clean copy). Finally, in the sub-section 3.6 we have clearly emphasized the potential of every selected package and function and they are working in our model. Regarding the function used for evaluating model performance (Leave-One-Out Cross-Validation), we have stressed that it is “*beneficial for small datasets, detects overfitting, and provides error estimates with comparatively good bias and variance properties [118]. In LOOCV, each data point is used once as a test set while the remaining data points form the training set. This technique ensures that every observation is tested exactly once, providing a comprehensive evaluation of model performance*” (see lines 357-365 of the clean copy).

However, regarding specific limitations of the methods used, we have preferred not to add specific details in the Materials and methods section. Anyway, we have added a sub-section in the Discussion called ‘5.3 Limitations of the methodological approach and improvements’, covering all the limitations and issues specifically associated to our results and findings, instead of highlighting general limitations of our methodology. We believe that this new section in the Discussion enhances the scientific value of the study, by critically examining the methods used. Moreover, focusing on limitations and critical aspects of the methodology in the Discussion allows us to link targeted improvements to limitations (see lines 719-745 of the clean copy).

***Ed_8: The "4. Results" section presents an extensive collection of data, but the excessive detail and lack of clear structure significantly hinder the identification of key findings. The results are described in a descriptive manner, without a clear connection to the study's objectives or emphasis on their importance in the context of erosion and sediment dynamics. The section lacks an analysis that would clarify the implications of the presented data. There is also insufficient critical reflection on potential measurement errors and methodological limitations, which diminishes the reliability of the results. In my opinion, better organisation of the section and a focus on the most important results and their significance are essential to improve its effectiveness.***

CA: Thank you for this comment. We have modified and rearranged the Results sub-sections in order to align them with the steps of the methodological framework (see Figure 2). Therefore, the structure of the Results section has been better improved, properly reflecting the study’s objectives, while the key findings have been highlighted. Furthermore, we have better emphasized the implications of the presented results, especially in the case of FTIR analysis results (sub-section 4.1), Random Forest model’s performance evaluation (sub-section 4.4), and Feature importance and variable interactions (sub-section 4.5).

However, we respectfully disagree with the claim that results would be presented in a descriptive way. Every sub-sections is presenting numerical results and outputs; for instance, we presented basic statistics for every analysed band of the FTIR absorption spectrum (see lines 384-388 of the clean copy), as well as for the FTIR indices (see lines 389-398). A new paragraph related to Spearman Correlation analysis, highlighting the implications and significance of these numerical values for our subsequent interpretation, has been added: “*Table 6 summaries the Spearman correlation coefficients among the calculated indices. Indices A/B and A/D are strongly correlated (i.e., 0.92), indicating that SOM hydrophobicity is well characterized in this study. The high correlation between A/D and C/D indices (i.e., 0.86) implies that hydrophobicity and relative carbonate content in the samples may be closely linked. However, even though the high correlation between B/D and C/D indices, their slightly lower coefficient value (i.e., 0.83) suggests that the two indices are capturing slightly different aspects of SOM-cation dynamics in the study area. The moderate correlation between A/B and C/D indices (i.e., 0.68), as well as the lower correlation between A/B and B/D indices (i.e., 0.55), suggests that the spatial distribution and accumulation of hydrophobic vs. hydrophilic SOM components might be driven by different landscape processes and dynamics (e.g., water fluxes, sediment erosion, transport and deposition) and/or environmental conditions*” (see lines 399-408). Results referred to Figure 5 are presented in a descriptive manner because they are related to the spatial distribution of the observed FTIR values across the watershed, thereby cannot be presented in numerical way (lines 411-431; please consider that these descriptive results are however really relevant for our interpretation; see Discussion).

Results related to datasets selected as feature variables have been rearranged and grouped in a single sub-section (4.2 Datasets selected as feature variables). Now we have clearly pointed out that “*these data represent a substantial set of environmental variables and watershed characteristics that potentially influence the geospatial variability of organic matter characterization indices used as potential indicators of erosion susceptibility*” (lines 453-455 of the clean copy). Please consider that basics statistics for the raster variables selected as feature variables have been moved to Supporting Information (S3 Appendix) in order to improve the general readability of the section and focus on the key findings. Results linked to lithological and LULC groupings have been preserved.

In sub-section 4.4, results of the statistical metrics evaluated in this study are presented in a numerical and schematic manner, emphasizing the statistical implications of such results for Random Forest model’s performance evaluation. We have finally concluded that “*Experiment 1 performed generally better than the other two experimental designs, demonstrating that the entire upstream hydrological contributing areas (Fig. 4a) provide the best way for investigating the interplay between SOM composition and selected feature variables in this landscape setting (Table 7)*” (lines 501-503).

In sub-section 4.5, results of the feature importance and variable interactions are presented in a schematic way, following the most common way for presenting such results in the scientific literature.

Please consider also that we have incorporated a new sub-section in the Discussion where we discuss the limitations of our methodological approach in relation to the results and findings (see lines 719-745 of the clean copy).

***Ed_9: The "5. Discussion" section in the manuscript presents an interpretation of the results, but it lacks consistency in both structure and analysis, which significantly undermines its scientific value. The authors largely focus on reiterating the results rather than engaging in their critical interpretation and situating them within a broader scientific context. There are insufficient references to relevant literature that could enable comparisons with other studies and evaluate the originality of the findings. In many parts, the section remains overly general, failing to address key questions about the significance of the results for understanding erosion processes and sediment dynamics under the specific conditions of a Mediterranean watershed. In its current form, the "Discussion" section appears disorganised and fails to fully capitalise on the potential of the findings, which diminishes its value as a critical component of the article. Improving the quality of this section requires better organisation, a stronger focus on analysing the results in both scientific and practical contexts, and the incorporation of a more critical perspective on their interpretation and limitations. Without these changes, the section does not fulfil its role as the foundation upon which the manuscript’s conclusions are built.***

CA: Thank you for this comment**. We have deeply enhanced the Discussion section by integrating new discussion/interpretation and reorganizing information**. Some information has been moved to other sections of the manuscript, e.g., results of the Spearman correlation analysis (see lines 399-408 of the clean copy). We have checked throughout the Discussion and removed/modified all information associated to reiterated results. We have added new references in order to link our findings to the broad scientific context and, in particular, to regional and local specificities. Moreover, we almost completely avoid the use of indices and technical terminology, referring directly to what these indices are representing (e.g., SOM relative hydrophobicity/wettability, cation exchange capacity, organic matter-cation associations). This should make the Discussion more fluent and accessible to potential readers.

**The Discussion has now been structured into three different sub-sections**, in order to better arrange the information and improve consistency: 1) **5.1 FTIR-derived indices as indicators of soil degradation and erosion processes**, 2) **5.2 Geospatial relationships between target and feature variables**, and 3) **5.3 Limitations of the methodological approach and improvements**.

In sub-section 5.1, we focus on interpreting the findings related to the role of the indices as indicators of soil degradation and erosion processes. Particularly, we focus on the most performing experiment for each index. Specifically, our interpretation relies on linking results of the Random Forest model to the processes occurring in the study area during the study period. Moreover, we have largely improved the interpretation by referring to the geomorphic and hydrogeological evidence from other areas in the same regional context during the same period, enhancing the scientific value of our interpretation: “*However, fluvial sediment collected during the sampling campaign appear to not reflect very well these processes (Fig. 1b). Thus, we hypothesize that this evidence might be linked to the type of erosion process captured in the sampling. Sheet erosion was likely the dominant erosion process during the heavy rainfalls occurred in the upper Val d’Arda in May 2023, pouring out sediment and organic matter from upland areas into the drainage system. Although the watershed experienced severe sediment supply into the rivers, the hydro-meteorological event occurred in May 2023 likely impacted less on geomorphic activity than many other watersheds in the same region [i.e., Emilia-Romagna; see 126-128]. Indeed, no significant landslide (re)activations, hydro-geological hazards, or floods were observed by the Authors or reported by local authorities in the watershed [see also 75,76]. Hence, considering that diffuse sheet erosion is often difficult to detect in the field or from aerial images, especially under tree canopy, areas affected by this process could not have been adequately covered in the maps used for Experiments 2 and 3 [62] (Figs. 4b, c). This might explain why this process is better represented in CAs of Experiment 1 (Fig. 4a), particularly in forested areas of the watershed (Fig. 7i)*” (see lines 575-587 of the clean copy). In addition, we have largely enhanced the interpretation of the findings related to Experiment 3, which was probably the most cryptic and less discussed part in the old version of the manuscript. Thus, we have added a new interpretation, as well as a new hypothesis and research perspective: “*Conversely, relative CEC is better predicted in hazard-prone hotspots affected by land degradation, characterized by relatively high geomorphic potential and sediment connectivity [62] (Experiment 3; Fig. 4c; Table 7). Hence, our results suggest that the B/D index could be a valuable proxy for modelling soil degradation by geomorphic activity in most vulnerable areas of the watershed. Such degraded areas are probably characterized by a reduced CEC, due to the degradation of topsoil rich in SOM and organo-mineral fine fractions caused by erosion and associated processes [129,130]. Other studies previously underscored a link between reduced CEC levels, low SOM content, and increasing erosion rates in Mediterranean environments, highlighting the critical role of organic matter for soil structural stability and erosion prevention [131,132]. From a certain point of view, this evidence imply that diffuse erosion processes might have affected areas already impacted by other geomorphic processes, as more susceptible to further degradation. However, this evidence appears to contrast with the results obtained from the other three indices (Table 7), as it suggests that hazard-prone hotspots played a more important role in sediment production and export during the study period [62]. As a possible explanation, we hypothesize that the organic matter analysed in the individual samples, whose signal is reflected in the calculation of the various indices (Table 1), may originate from diverse areas within the respective CA. Soluble Dissolved Organic Matter (DOM) might have a different origin influenced by various factors and may have followed distinct pathways compared to organic matter in particulate form and/or associated with sediments, while the latter could have been transported differentially based on the characteristics of soil particles [133]. Moreover, Berhe [134] observed that the decomposition rates of organic matter differ considerably along dynamic toposequences and across soil depth gradients, where the effect of transfer of topsoil and associated SOM from eroding to depositional landforms, as well as of its burial by subsequent erosion events, depends on local environmental conditions. This could have implications for the role of CEC proxy as potential indicator of land degradation as, generally, the greater the degree of decomposition of the SOM, the higher the CEC of the SOM [135]. Nadeu et al. [136] already demonstrated how different land use and geomorphic settings can affect sediment sourcing and SOC dynamics in Mediterranean watersheds. Nevertheless, this study was not aimed at conducting a detailed molecular chemical analysis of organic matter to determine its origin within the watershed, nor at performing a pedological analysis to investigate soil properties in relation to degradation dynamics. Thus, the approach employed in this study is unable to offer more detailed insights into the observed relationships. However, these preliminary findings serve as a crucial starting point for further in-depth studies*” (see lines 588-616).

In the sub-section 5.2, we focus on the geospatial relationships between target (FTIR indices) and feature variables (environmental variables). This part has been improved by reorganizing information and give more value to both generic and specific aspects related to the Mediterranean context. New references have been incorporated. Most of the information underlined in the interpretation directly links to the state-of-the-art provided in the Introduction, further stressing and elaborating on the association between the SOM indices and degradation/erosion susceptibility. Particular attention has been paid to the Mediterranean environment, e.g., “…*in many Mediterranean watersheds such as the upper Val d’Arda, clay-rich soils can experience intensified shrinking-swelling phenomena which enhance soil cracks under dry conditions [47,142,143]. Bosino et al. [144] highlighted that after long dry periods, these cracks may allow huge water infiltration into the upper topsoil horizon, leading to a subsequent closure of the fissures in the surface crust. During moist conditions the clay rich substrate enhance rapid runoff processes due to reduced infiltration rates, especially in shallow soil profiles with minimum storage capacities [144]. However, structural weaknesses in high-hydrophobic soils can create preferential flow paths [145], leading to uneven wetting, aggregate breakdown, and increased erosion rates under heavy rainstorms. Nevertheless, other studies pointed out that high organic matter content can stabilize clays reducing swelling and crack formation [146]. Therefore, our findings might have relevant implications for Mediterranean watersheds similar to the upper Val d’Arda, where clay content in soils ranges from 7 to 47% (Fig. 7c), with a mean value of ~ 30% (cfr. S3 Appendix)*” (see lines 637-648). And again: “*as with many other watersheds in the Northern Apennines, in the study area the past and current riverbed evolution is a function of fluvial morphodynamics, as well as lithological and structural characteristics of the landscape [61]. La Licata et al. [47] emphasised that in the lower part of the upper Val d’Arda, where gentle open slopes composed of pelitic and chaotic rock formations prevail and the valley bottom is wider, slope and fluvial sediments can be easily stored in depositional storages. According to Doetterl et al. [150], these findings underscore the importance of adopting a landscape-based modelling approach for assessing the complexity of SOM dynamics and the relations with their shaping factors, informing useful predictions of organic matter cycling and soil carbon sequestration in degraded watersheds*” (lines 682-690). In general, we have further stressed the implications for erosion susceptibility and organic matter-sediment dynamics throughout the Discussion.

In sub-section 5.3, we have incorporated a section covering limitations of the methodological approach and improvements, further enhancing the critical analysis already included in the old version of the manuscript (see lines 719-745).

***Ed_10: The "6. Conclusion" section, in its current form, fails to serve as a concise summary of the manuscript and the findings derived from the research. While the authors attempt to articulate the key conclusions, these lack clarity, precision, and a clear connection to the primary objectives of the study. Rather than providing a succinct synthesis of the most important findings, this section appears to repeat content presented in other parts of the manuscript, rendering its added value minimal.***

***Another significant issue is the absence of a clear emphasis on how the study’s findings contribute to addressing the research problem or expand knowledge on erosion processes and sediment dynamics within the specific environmental context of the investigated watershed. This lack of focus further detracts from the section’s effectiveness as a meaningful conclusion to the manuscript.***

***Therefore, I believe that the "Conclusion" section requires a thorough revision to concisely yet comprehensively summarise the key findings, highlight their significance in both scientific and practical contexts, and address the study's limitations. In its current form, this section is overly general, lacks clear focus, and fails to fulfil its role as an effective conclusion to the manuscript.***

CA: Thank you for this comment. The Conclusion section has been totally modified and adjusted, closely following your comments (see lines 747-791). According to your considerations, we really believe this new version of the Conclusion fulfil its role as an effective conclusion to the manuscript.

***Ed_11: The "Acknowledgments" section includes links to online resources, which appears inappropriate as it raises concerns about the durability of such references and gives the section a less formal character.***

CA: Thank you for this comment. We have removed links to online resources. Moreover, we have removed all information already provided in the Funding section, according to the submission guidelines.

***Ed_12: Importantly, according to the journal's guidelines, in-text citations should be numbered sequentially in the order they appear and enclosed in square brackets, e.g., "[1]." Deviation from this required format suggests a lack of diligence in aligning the manuscript with the journal's requirements. Improper citation formatting complicates the editorial and review process; therefore, it is crucial to adapt the references to comply with PLOS ONE’s standards.***

CA: Thank you for this comment. We have modified accordingly.

Sincerely,

Manuel La Licata

(*Corresponding Author*)
