# Supplementary material for: FTIR-derived soil degradation indices and stochastic modelling of organic matter–sediment dynamics in a Mediterranean watershed: A Northern Apennines case study
Source: PLoS One. 2025 Aug 21;20(8):e0330252. doi: 10.1371/journal.pone.0330252 (PMC12370054; doi:10.1371/journal.pone.0330252)
Supplement: Attachment — Submitted filename: Response to Reviewers.docx [file pone.0330252.s006.docx]

Dear Editor,

Dear Reviewers,

We would like to express our gratitude for your valuable comments and suggestions, which have significantly contributed to improve the manuscript. We have tracked the changes throughout the manuscript and provided a clean copy without tracked changes, as requested by the journal. We have addressed your requests and comments throughout the manuscript. Please find below our responses. The specific references to the modified lines are referred to the “clean copy”.

**Reviewers’ comments:**

***Reviewer_1: The title of the article suggests research in the Mediterranean region, whose coastline is very long, extensive, and is based on many countries, regions, etc. Meanwhile, when reading the text, we read that the authors are basically studying the catchment area of one river flowing into the Adriatic Sea, which concerns the region of Emilia-Romagna, northern Italy, the Arda River. This means that the title should be changed, precisely defining the area.***

Corresponding author (CA): Thank you so much for the valuable comment. We agree with you and modified the title accordingly. Now the title sounds: “*Integration of FTIR-based soil degradation indices with stochastic modelling to assess spatial patterns of organic matter-sediment dynamics in a Mediterranean watershed – A case study from the Northern Apennines*”. We believe that this title better reflect the regional specificity of the study watershed, while keeping the attention of the reader to the Mediterranean. Hence, we believe that the reader should be clearly informed from the title that we are dealing with Mediterranean characteristics, in spite of we are studying only one watershed in the Apennines. The title is 183 characters without spaces or 211 with spaces, thereby being consistent with journal requirements (i.e., max 250 characters).

***Reviewer_2: Fig. 1. The quality of these images is low, it needs to be improved. The hatching also raises concerns because the maximum height of 1300m is marked as if it were a height of over 5000m.***

CA: We greatly appreciate your comment and your attention to the quality of the figures. It is possible that the version of the figure displayed in the submission document does not show the overall map and the photographs below with optimal clarity. However, we have provided all images at a resolution of 500 dpi, and we are confident that the final publication will ensure high-quality rendering. If by “image quality” you referred to something else, we kindly ask you to clarify this point…

Figure 1 provides all the necessary elements to properly outline the study area in its geographical context, offering both a regional overview and a basic characterization of the topography of the area. This is intended to help the reader better understand the distribution of sampling sites across the landscape. A zoomed-in panel further illustrates the relative position of the samples along the valley floor, accompanied by representative photographs (in high resolution). Therefore, we believe that this figure offers a useful and essential tool for contextualizing the sampling design within the studied watershed. The legend clearly shows the elevation range of the study area, leaving no particular ambiguity about the topographic configuration or actual elevation of the watershed. The DTM was classified using graduated colours, based on a scale bar that is commonly used for elevation data, and semi-transparently overlaid on a hillshade map to improve the visualization of landscape morphology (an approach widely used for this type of map). Thus, we understand that the original colour scale, from light-blue (valley floors) to white (main peaks), may have been somewhat misleading at first glance, as it may have suggested higher alpine-like peaks. For this reason, we have adjusted the scale bar by removing the white colour, making the visualization more appropriate for the actual elevation range of the study area. Please check the new Figure 1 provided along with the revised submission. Anyway, please consider that Fig. 1a has been changed to accomplish journal requirements (see comment below; cfr. comment ‘Journal_6’).

***Reviewer_3: Line 134 and others: I suggest writing Roman numerals with a capital letter.***

CA: Thank you for the comment. Roman numerals have been rewritten using capital letters throughout the text.

***Reviewer_4: The authors described the catchment area of the studied river, also showing the lithology of the bedrock. They indicate in the text that mass movements occur there. Maybe it would be worth including a geological and soil map in this place? It would be good to explain why mass movements occur, whether it is the effect of soil instability resulting from heavy rains, the arrangement of rocks in the subsoil, or improper use of soils. This information has an impact on the rest of the text, especially since the authors themselves indicate that mass movements destroy soils (line 149). It is also worth considering how fields are ploughed, which can facilitate or hinder surface runoff of water on slopes.***

CA: Thank you for your suggestion and consideration. In our opinion, including geological and soil maps in the Study Area chapter would go beyond the scope of this work, considering that this study does not aim to explore relationships between erosion and landslide processes and specific variables such as all the outcropping geological formations or soil types. In this study, geological formations are grouped based on lithological characteristics and relevant/available soil properties are taken separately as single variables. Moreover, for the whole study area, the available soil map is at a scale of 1:250,000, while more detailed information (1:50,000) is only available for limited parts of the watershed. Therefore, the integration of pedological data in this form would likely not be particularly useful. The core of the study lies in the relationship between the so-called ‘target variables’ (FTIR indices) and the ‘feature variables’, which are analyzed within a machine learning framework rather than from a qualitative perspective. All lithological and pedological features that are relevant and available in an appropriate format (e.g. raster, shapefile) have already been incorporated into the model, independently from the classifications found in soil maps. Thus, we believe that integrating geological or soil maps would not contribute substantially to the analysis, since these variables are already considered in the model and are presented as part of the results, particularly in Figure 7, which already shows the spatial distribution of lithological, pedological, and land use data.

However, we agree that adding some further contextual information in the Study Area chapter could help clarify key aspects of the area's geomorphology. Therefore, we have revised the part referring to landslides as follows: “*Landslides are the dominant land degradation process, exhibiting considerable variability in magnitude and frequency. In the study area, landslide activity is largely dominated by periodic reactivation of pre-existing large-scale landslide bodies, primarily triggered by intense or prolonged rainfall events. Fluvial undercutting at the toe of landslide deposits also plays a critical role in destabilizing valley slopes*” (lines 153-157).

Regarding the suggestion to provide information on how the fields are ploughed, we unfortunately do not have detailed data on this. Most of the cultivated areas in the study area are classified as rainfed arable lands, with very few hectares of vineyards and orchards (they are not enough to justify a separate class as they are very small and sporadic polygons in the overall land use map). Please note that the variable ‘cultivated fields’ is among the feature variables integrated into the model, but the results did not reveal any significant relationship between this variable and the FTIR indices. Please also note that in the Study area chapter, we explicitly stated that: " *Rainfed arable lands represent the predominant agricultural landscape in the watershed*..." (line 168).

***Reviewer_5: Table 3 is interesting, but the authors could include photographs of typical examples of rocks and a map of agricultural use of the research area.***

CA: Thank you for your appreciation. In our opinion, adding photographs of representative rock types could be somewhat interesting, but it would not substantially add value to the research, as the characteristics of the lithological groups are already clearly described in Table 3. Moreover, this information is relevant to our study because it relates to the parent material of the soils. Additionally, the characteristics of sediments may be affected by the bedrock from which they come from, rather than to rock outcrops or lithological material themselves. Therefore, including pictures of rocks or outcrops may not be entirely relevant in this context. Regarding agricultural land use in the study area, a classification comprising 10 land use classes (including agricultural areas) has already been integrated into the model. A map showing the classified land use is also presented as part of the results in Figure 7.

***Reviewer_6: After Fig. 5, the authors describe Figure 7 (line 439) in the text, so it should be moved so that the order of citation of graphics in the text is maintained. There is no description of Figure 6 in this paragraph. This needs to be supplemented. There is only one reference in line 473, but it is too little for the information contained there.***

CA: Thank you for your comment. We understand the point you raised. However, Figure 6 was already cited at line 451 (previously line 436 in the former submitted version), before the citation of Figure 7 at line 454 (previously line 439). Therefore, the consistency in the figure numbering and citation order is maintained. Also, we would like to clarify that the textual information regarding basic statistics of the raster-based feature variables (Figures 6 and 7) is not reported in the manuscript text but only in the Supporting Information, as explicitly stated in the manuscript (lines 454-455). This was a specific request from the Editor during the initial revision round, before the manuscript was sent out for peer review. Indeed, the Editor specifically asked us to move all numerical and technical data (such as these basic statistics) to the Supporting Information, in order to avoid overloading the main text with non-essential content and to help readers focus on the most relevant information for interpretation and discussion. On the other hand, the subsequent information referring to Figure 7 (lines 456-467) mainly concerns the shapefile-based variables (i.e., lithology and land use), from which proportions were extracted. In this case, the text does not only present numerical data that could be moved to the Supporting Information, but also provides qualitative and descriptive insights into the distribution of these variables across the study area. These elements are indeed important to support the discussion (you will see that Figs. 7h and 7i have been extensively cited in the discussion).

***Reviewer_7: Line 571, requires consideration, the hydrophobicity of matter alone does not have to stop erosion, which can be caused by, for example, soil instability and mechanical washing out. In addition, chemical compounds can appear in polluted waters that cause emulsification of such compounds.***

CA: Thank you for your comment. However, in the discussion, we avoid any reference to a "protective" or "preventive" role of hydrophobicity against soil erosion. On the contrary, we stated the pedological significance of hydrophobicity on soil erosion in the Introduction, before outlining the objectives of the present work. We are fully aware that erosion can occur in areas affected by soil instability, which is the reason why we decided to focus on three different hydrological/geomorphological settings that take these aspects into account. All of this is addressed in the Discussion (see paragraphs below).

**--- --- --- --- --- --- --- --- --- --- --- --- --- --- --- --- --- --- --- --- --- --- --- --- --- --- --- --- --- ---**

**Editor’s additional comments:**

***Editor_1: In addition to the reviewers' comments, I would like to draw your attention to several further issues that require careful consideration. While your integration of FTIR-derived indices with Random Forest modelling is methodologically sound, the interpretation of these proxies as direct indicators of erosion susceptibility appears overstated. The manuscript lacks empirical validation linking the FTIR indices to actual erosion measurements, sediment yields, or field-observed processes. This disconnect should be addressed with a more cautious interpretation of your findings, clearly acknowledging the proxy-based and indirect nature of your assessments.***

CA: Thank you so much for the comment. We agree with your point of view. We have added a short paragraph in the section ‘5.3 Limitations of the methodological approach and improvements’: “*In general, the significance of these findings relies on the assumption that the FTIR indices used in this study can serve as proxies for soil hydrophobicity, CEC, and organic matter–cation interactions (Table 1) within this environmental context, as supported by evidence drawn from the literature [39,50-55,84]. The obtained results represent an initial preliminary attempt towards a better understanding of such relationships. However, these results should be interpreted with caution, e.g., as we discussed for their relevance in terms of erosion processes. That is, an empirical evaluation linking FTIR indices to actual erosion measurements—necessary to establish these indices as direct indicators—is currently lacking. Nonetheless, additional studies are needed to explore more detailed relationships between these potential FTIR-based indicators and the chemical and pedological characteristics of sediment source areas*” (Lines 737-745). Moreover, we would like to stress the fact that in the manuscript we are frequently referring to these indices as “**potential** indicators”, thereby implicitly reflecting their nature in relation to the methodology and the approach employed in the study. In any case, in the manuscript we never refer to definitive evidence or unquestionable results, but we consistently use modal verbs (e.g., could, might, may, etc…). In this regard, we have slightly refined some sentences (please check the Tracked Changes Copy).

***Editor_2: Moreover, your conclusions frequently extrapolate results from a relatively small catchment in the Northern Apennines to “Mediterranean watersheds” in general. Such generalisations are not sufficiently substantiated and should be carefully delimited to the specific physiographic and climatic context of your study area. A more balanced formulation that frames your findings as case-study insights rather than general regional conclusions is recommended.***

CA: Thank you for the valuable comment. We agree that, in the Conclusion, we should refer to these extrapolations more cautiously. Therefore, we have made some corrections:

- Lines 785-786: we have clearly specified that we selected a “*temperate agricultural-forested area of the Northern Apennines chosen as representative of Mediterranean environments*”;
- Lines 802-803: “*This underscores the complexity of SOM dynamics in environments with similar characteristics and supports*…”;
- Lines 810-812: “*These findings carry significant implications for Mediterranean watersheds having similar vegetation, as well as for regions with seasonally contrasting climates and lithologies dominated by weak, stratified, clay-rich rocks*”;
- Lines 816-818: “*These insights suggest that such landscape-based modelling could be a valuable approach for predicting organic matter cycling and carbon sequestration in complex, dynamic Mediterranean watersheds with similar landscape configuration*”.

***Editor_3: Another aspect requiring attention concerns the sampling strategy. Given that samples were collected following a significant rainfall event, it would be pertinent to discuss how this may have influenced sediment composition and whether such conditions are representative of the typical sediment dynamics in the catchment. This context is essential for interpreting your results.***

CA: Thank you for the comment. In the section ‘3.2 Sampling of fluvial sediments’ we provided a detailed sampling strategy. We stated that we selected only freshly deposited river sediments collected shortly after the heavy rainfall event (lines 211-212), further providing the criteria through which these kind of sediments were identified in the field. Moreover, we stated that this uniformity in deposition conditions among the samples is needed to maintain high organic content and preserving biochemical properties (lines 216-218). These requirements are frequently used also in sediment fingerprinting studies (see also the cited literature). Additionally, by referring directly to our sampling strategy, we wrote that sediments deposited under similar hydrodynamic conditions generally exhibit more consistent physical and biochemical properties, which is crucial for comparative studies (lines 220-221). In addition, in the same section we have added a new sentence clearly stating our assumption, which is indeed essential for many of the interpretations illustrated in the Discussion section: “*Based on these considerations, we assume that the differences observed in the sampled material (Fig. 1) can be attributed to a set of environmental variables that influenced the chemical composition of SOM from its origin in the upstream areas to its deposition at the sampling sites*” (lines 221-224). Finally, there’s one more point related to your comment that needs to be addressed, although it is mostly referred to the sampling preparation for FTIR analysis. That is, in the section ‘5.3 Limitations of the methodological approach and improvements’ we have included an additional consideration: “*[…] if the organic component is not separated from the mineral fraction, accounting for the particle size effect and effective organic matter content on organic matter–sediment interactions and the detectability of SOM characteristics could reveal additional insights into the variability of the measured band intensities [49,151]. This might be of particular interest whether the sediment’s particle size distribution and/or organic constituents allow for hypotheses regarding the origin of the sediment within the watershed or enable inferences about the conditions and the selectivity of the mechanism responsible for its erosion and transport [78, 79, 152, 153]*” (lines 754-760). Please note that we have also added some references to give more value to the argument (i.e., 151, 152, 153; the last part of the reference list has consequently changed).

***Editor_4: Additionally, while your Random Forest models are well described, there is little discussion of model uncertainties, potential overfitting, or limitations. Providing a critical reflection on the robustness of your models and discussing the possible influence of dataset characteristics would enhance the scientific transparency of your work.***

CA: Thank you for the comment. We have added an additional paragraph to the section ‘5.3 Limitations of the methodological approach and improvements’: “*Despite the reliability afforded by the LOOCV and ensemble structure of RF model, residual uncertainties may persist due to potential imbalances in the datasets, the underrepresentation of specific geomorphic features where geomorphological mapping is limited by a dense forest cover, or variability in SOM composition that is not captured in the sampling. These factors may limit the model’s transferability to unsampled areas having diverse environmental conditions*” (lines 771-775). Anyway, please consider that some information was already given in the same section of the Discussion, especially regarding the RF model. Moreover, in the Materials and Methods section these aspects are already provided. For instance, in lines 366-370 we stated that: “*The RF algorithm, which is an ensemble machine learning method [56], was then used to model the relationships between the selected features and target variables. RF operates by constructing multiple decision trees during training and outputting the mean prediction of the individual trees. It excels in handling complex and non-linear relationships by averaging the results from numerous trees to improve accuracy and control overfitting*”. Finally, in the lines 372-374 we stated that “*To evaluate model performance, we implemented Leave-One-Out Cross-Validation (LOOCV) on the entire dataset as it is beneficial for small datasets, detects overfitting, and provides error estimates with comparatively good bias and variance properties [118]*”.

***Editor_5: Finally, the integration of lithological controls and the role of mass movements in sediment connectivity and SOM dynamics are not fully explored. Although mentioned in the text, these factors are not quantitatively assessed nor sufficiently linked to your FTIR-based analyses. A more detailed examination of how different lithologies and geomorphological processes affect your observed patterns would significantly strengthen the study.***

CA: Thank you for your comment. In general, a detailed examination of the integration of lithological controls and the role of mass movements in sediment connectivity and SOM dynamics, as well as a quantification of these factors, goes beyond the scope of the present work. It is true that, in the Study Area chapter, we mentioned that the watershed is significantly affected by landslide activity, which characterizes its geomorphological setting. However, in the interpretation and discussion of the results, we focused solely on the evidence observed during the study period and, in particular, on the variables that most significantly influence the model (i.e., the top 10 most significant feature variables, as explained in the Materials and Methods section). On the one hand, in the first part of the Discussion, we explicitly clarify that no landslides or other high-magnitude geomorphic processes were recorded or observed in the study area during the investigation period. This fact, combined with a high observed sediment yield, allowed us to interpret the results as primarily associated with diffuse surface erosion like sheet erosion (see lines 591–598). Therefore, emphasizing mass movements and the relations with lithological controls in the discussion would be misleading for the reader and deviate from the main focus of the study.

However, mass movements are considered in our model because geomorphological areas included in Experiment 2 and the hazard-prone areas in Experiment 3 are indeed affected by such soil degradation processes (among others), which is clarified in the text (lines 587–590). In this regard, we have added a clarification regarding Experiment 3: “*These hazard-prone areas mostly include landslides highly connected to the drainage system and overlaid by additional processes like rill-interrill erosion, gullying, stream incision, and bank erosion, as well as steep rock walls affected by rockfalls and debris flows*” (lines 332-334). Moreover, in the Discussion, we previously addressed the role of highly active sediment sources in the interpretation of the B/D index. In this regard, we have further modified the text to include a more explicit reference to landslides, according to your suggestion: “*Even though the influence of Elevation is more or less relevant in all experiments (Fig. 4; cfr. Feature Importance plots in Supporting Information), the peculiar effect revealed by Experiment 3 is likely related to the distribution of geomorphologically highly active areas (e.g., landslide deposits connected to the drainage system and affected by additional processes like surficial soil erosion and/or stream incision, and steep rock walls affected by rockfalls and localized debris flows) with respect to the elevation gradient (Fig. 11; Fig. 4c) [see 62]. In other words, highly active sediment sources and eroding steep slopes, mostly distributed at medium-high elevations, likely retain less SOM rich in C=O groups because the export of organic matter is higher and the accumulation of decomposed compounds is limited [134]*” (lines 706-714).

Regarding lithological variables more specifically, in the interpretation and subsequent discussion of the results we focused exclusively on the most influential predicting variables identified by the model, as previously mentioned. From this perspective, the role of lithological factors was clearly addressed by discussing the influence of carbonate-rich bedrocks and high clay content, which is to some extent determined by the underlying bedrock.

**--- --- --- --- --- --- --- --- --- --- --- --- --- --- --- --- --- --- --- --- --- --- --- --- --- --- --- --- --- ---**

**Journal Requirements:**

***Journal_1: Please ensure that your manuscript meets PLOS ONE's style requirements, including those for file naming.***

CA: Ok, done.

***Journal_2: Please note that PLOS ONE has specific guidelines on code sharing for submissions in which author-generated code underpins the findings in the manuscript. In these cases, we expect all author-generated code to be made available without restrictions upon publication of the work. Please review our guidelines at https://journals.plos.org/plosone/s/materials-and-software-sharing#loc-sharing-code and ensure that your code is shared in a way that follows best practice and facilitates reproducibility and reuse.***

CA: Ok. We have made the Random Forest code used for our experiments available at the following link (GitHub): <https://github.com/Odunayo3/SoilDegradationModeling.git>. The link is already published. The link has been attached in the Supporting Information file accompanying the manuscript.

***Journal_3: In your Methods section, please provide additional information regarding the permits you obtained for the work. Please ensure you have included the full name of the authority that approved the field site access and, if no permits were required, a brief statement explaining why.***

CA: Ok, done. The following statement has been added in the first section of the Materials and Methods section: “*Access to the study area and sampling sites did not require any permission from local authorities, as the watershed is an open-access area, and no private property was violated in any way*” (lines 189-190).

***Journal_4: Please state what role the funders took in the study. If the funders had no role, please state: "The funders had no role in study design, data collection and analysis, decision to publish, or preparation of the manuscript." If this statement is not correct you must amend it as needed. Please include this amended Role of Funder statement in your cover letter; we will change the online submission form on your behalf.***

CA: Ok, I have included the Role of Funder statement in the cover letter: “*The funders had no role in study design, data collection and analysis, decision to publish, or preparation of the manuscript*”.

***Journal_5: When completing the data availability statement of the submission form, you indicated that you will make your data available on acceptance. We strongly recommend all authors decide on a data sharing plan before acceptance, as the process can be lengthy and hold up publication timelines. Please note that, though access restrictions are acceptable now, your entire data will need to be made freely accessible if your manuscript is accepted for publication. This policy applies to all data except where public deposition would breach compliance with the protocol approved by your research ethics board. If you are unable to adhere to our open data policy, please kindly revise your statement to explain your reasoning and we will seek the editor's input on an exemption. Please be assured that, once you have provided your new statement, the assessment of your exemption will not hold up the peer review process.***

CA: Ok. The three datasets of ‘target’ and ‘feature variables’ related to the three experiments, along with the raster and shapefile data of the feature variables, the shapefile of the contributing areas for each experiment, and a shapefile with the location of the sampling sites, are available at the following link: <https://doi.org/10.5281/zenodo.15097417>. The link has been attached in the Supporting Information file accompanying the manuscript. The data will be published in Zenodo after acceptance.

***Journal_6: We note that Figures 1 and 4-7 in your submission contain map/satellite images which may be copyrighted. All PLOS content is published under the Creative Commons Attribution License (CC BY 4.0), which means that the manuscript, images, and Supporting Information files will be freely available online, and any third party is permitted to access, download, copy, distribute, and use these materials in any way, even commercially, with proper attribution. For these reasons, we cannot publish previously copyrighted maps or satellite images created using proprietary data, such as Google software (Google Maps, Street View, and Earth).***

CA: Regarding the figures, none of the base maps used in the manuscript are derived from Google products or proprietary data sources (e.g., Google Maps, Street View, Earth). For Figure 1, data attribution has been provided directly in the figure caption in the revised version of the manuscript. The attributed data are subject to a CC BY 4.0 license and are in the public domain. Base Map of Fig. 1a has been removed. For other base maps, please refer to the following official links, where the type of license and additional information are reported: 1) Fig. 1b - <https://metasfera.regione.emilia-romagna.it/ricerca_metadato?uuid=r_emiro:2016-08-08T155835>; 2) Fig. 1c - <https://metasfera.regione.emilia-romagna.it/ricerca_metadato?uuid=r_emiro:2017-11-24T113154>. For Figure 4, the base map consists of a digital orthophoto freely provided by the Emilia-Romagna Region through the official geoportal and is classified as ‘public data’ (cf. <https://metasfera.regione.emilia-romagna.it/ricerca_metadato?uuid=r_emiro:2022-03-11T113115>). Therefore, it is not based on Google or similar sources. Attribution has also been included in the corresponding caption. Figures 5 and 6 only contain original results from this study (with all materials and data sources clearly described and referenced in the relevant section of the paper), and no base maps have been used. Figure 7 contains data derived from pre-existing datasets provided by the regional authorities, as clearly explained in the Materials and Methods. All these datasets are provided by the Emilia-Romagna Region under the CC-BY-4.0 license (see all the following links where the type of license is highlighted). In particular, Fig 7a depicts the SOC stock (<https://datacatalog.regione.emilia-romagna.it/catalogCTA/dataset/r_emiro_2023-08-09t162508>); Fig. 7b depicts the SOC % (<https://datacatalog.regione.emilia-romagna.it/catalogCTA/dataset/r_emiro_2023-08-09t160758>); Figs. 7c, d, e, f depict the Clay %, Silt %, Sand %, and Skeleton %, respectively (<https://datacatalog.regione.emilia-romagna.it/catalogCTA/dataset/r_emiro_2023-08-02t140310>); Fig 7g depicts the pH (<https://datacatalog.regione.emilia-romagna.it/catalogCTA/dataset/r_emiro_2023-08-02t144815>); Fig 7h includes data derived from the geological unit dataset (<https://datacatalog.regione.emilia-romagna.it/catalogCTA/dataset/r_emiro_2017-06-12t113543>); Fig 7i includes data derived from the Land Use dataset (<https://geoportale.regione.emilia-romagna.it/catalogo/dati-cartografici/pianificazione-e-catasto/uso-del-suolo/layer-9>). All these datasets were cited and acknowledged in the Materials and Methods as well as in the Reference list.

***Journal_7: Please review your reference list to ensure that it is complete and correct. If you have cited papers that have been retracted, please include the rationale for doing so in the manuscript text, or remove these references and replace them with relevant current references. Any changes to the reference list should be mentioned in the rebuttal letter that accompanies your revised manuscript. If you need to cite a retracted article, indicate the article’s retracted status in the References list and also include a citation and full reference for the retraction notice.***

CA: Ok, done. The reference list is complete and correct. We have slightly changed it starting from Reference n. 151 in order to include additional literature. To the knowledge of the Authors, no retracted papers were included.

***Journal_8: While revising your submission, please upload your figure files to the Preflight Analysis and Conversion Engine (PACE) digital diagnostic tool, https://pacev2.apexcovantage.com/. PACE helps ensure that figures meet PLOS requirements***.

CA: Ok, done. All the figures have been processed through the PACE tool and adjusted accordingly.

Sincerely,

Manuel La Licata

(*Corresponding Author*)
