# Supplementary material for: FTIR-derived soil degradation indices and stochastic modelling of organic matter–sediment dynamics in a Mediterranean watershed: A Northern Apennines case study
Source: PLoS One. 2025 Aug 21;20(8):e0330252. doi: 10.1371/journal.pone.0330252 (PMC12370054; doi:10.1371/journal.pone.0330252)
Supplement: Attachment — Submitted filename: Response_to_Reviewers_auresp_2.docx [file pone.0330252.s007.docx]

Dear Dr. Przemysław Mroczek,

We would like to thank you very much for all the work you have done with us in these months, during several revision processes, which has led to a result that we are very satisfied. Please find below our responses.

**Editor’ comments:**

***Editor_1: Firstly, while your current title correctly reflects the study’s scope and regional focus, it is rather long and includes redundant wording that makes it unnecessarily complex. PLOS ONE encourages concise titles that convey the essential elements without superfluous phrases. I therefore strongly recommend shortening it to the following form: “FTIR-derived soil degradation indices and stochastic modelling of organic matter–sediment dynamics in a Mediterranean catchment: a Northern Apennines case study.” This version preserves all key terms but removes the overly extended structure and the phrase “to assess spatial patterns”, which is implicit in your methods and discussion.***

Corresponding author (CA): Thank you very much for the comment and the suggestion. We have decided to adopt the title you proposed, with just one word changed. We chose to keep the term “watershed” instead of “catchment” for consistency with the terminology used in the text. The title now reads as follows: "*FTIR-derived soil degradation indices and stochastic modelling of organic matter–sediment dynamics in a Mediterranean watershed: a Northern Apennines case study*".

***Editor_2: Secondly, I encourage you to refine your set of keywords to avoid repeating phrases already present in the title. For example, “Organic matter indices”, “Mediterranean watershed”, and “Geomorphological modelling” duplicate core title terms and thus reduce the added value of the keywords for search engines and indexing. A more effective set could include terms that highlight important aspects of your study not directly stated in the title. I suggest the following as an example: Soil erosion risk, FTIR spectroscopy, Soil organic matter composition, Random Forest modelling, Watershed connectivity, and Sediment transport processes. This selection emphasises the main processes, analytical approach, and spatial system interactions central to your manuscript.***

CA: Thank you very much for your comment and suggestion. Also in this case, we decided to use essentially the keywords you proposed, with only minor adjustments. Now the keywords are: “*Soil erosion susceptibility*, *FTIR spectroscopy*, *Soil organic matter composition*, *Random Forest modelling*, *Landscape connectivity*, *Sediment transport processes*”. We chose to avoid the term “risk” because in applied geomorphology this term has a specific meaning that does not apply to the scope of our work. Therefore, we would prefer to avoid potential misunderstandings among specialized readers. Moreover, compared to your proposal, we decided to keep “landscape connectivity” instead of “watershed connectivity,” as this term (and its related definition) is more relevant and consistent with the literature cited in the manuscript.

**Journal’s requirements:**

***Journal_1: Figures 1 and 5: The captions for these figures include appropriate attribution and therefore require no further changes.***

CA: Ok, thank you.

***Journal_2: Figures 6 and 7: Please can you update the captions of these figures to include attribution indication of the source of your map's data.***

CA: Ok, done. The two captions have been modified as follows.

- Fig. 6. Terrain variables of the upper Val d’Arda-Mignano watershed derived from GIS-based terrain analysis: (a) Elevation, (b) Flow Accumulation, (c) Slope, (d) Aspect, (e) Profile curvature, (f) Tangential curvature, (g) General curvature, (h) Total curvature, (i) Downslope Distance Gradient, (j) SAGA Wetness Index, (k) Terrain Ruggedness Index, (l) Stream Power Index, (m) Channel Network Base Level, (n) Vertical Distance to Channel Network. Variables (a), (c), (d), (j), (k), (m), and (n) are displayed in the full range. Variables (b) and (l) are displayed by applying a stretching factor of 0.5 standard deviations for a better visual representation. Variables (e), (f), (g), (h), and (i) are displayed by applying a stretching factor of 2.5 standard deviations. Data source: all the maps are derived from the Digital Terrain Model 5x5, Ed. 2014 (© Archivio Cartografico, Regione Emilia-Romagna; https://metasfera.regione.emilia-romagna.it/ricerca_metadato?uuid=r_emiro:2016-08-08T155835). Maps produced using the SAGA software (version 8.1.1).
- Fig. 7. (a-g) Soil properties of the upper Val d’Arda-Mignano watershed: (a) SOC stock, (b) SOC %, (c) Clay %, (d) Silt %, (e) Sand %, (f) Skeleton %, (g) pH, (h) lithological groups, and (i) LULC types. All the variables are displayed in the full range. (h) Lithological groups (Table 3): LI_1, Silicified Calcilutites and Silty Clays; LI_2, Varicoloured Clays and Shales; LI_3, Carbonate Turbidites; LI_4, Ophiolitic/Sedimentary Breccias and Olistoliths; LI_5, Arenaceous-Pelitic Turbidites group. (i) LULC types (Table 4): LU_1, Coniferous forests; LU_2, Beech forests; LU_3, Oak, hornbeam and chestnut forests; LU_4, Riparian vegetation; LU_5, Cultivated fields; LU_6, Anthropic areas; LU_7, Bare surfaces/Rocky outcrops; LU_8, Sparse vegetation in evolution; LU_9, Meadows; LU_10, Bushes and shrubs. Data sources: (a) https://datacatalog.regione.emilia-romagna.it/catalogCTA/dataset/r_emiro_2023-08-09t162508; (b) https://datacatalog.regione.emilia-romagna.it/catalogCTA/dataset/r_emiro_2023-08-09t160758; (c, d, e, f) https://datacatalog.regione.emilia-romagna.it/catalogCTA/dataset/r_emiro_2023-08-02t140310; (g) https://datacatalog.regione.emilia-romagna.it/catalogCTA/dataset/r_emiro_2023-08-02t144815; (h) https://datacatalog.regione.emilia-romagna.it/catalogCTA/dataset/r_emiro_2017-06-12t113543; (i) https://geoportale.regione.emilia-romagna.it/catalogo/dati-cartografici/pianificazione-e-catasto/uso-del-suolo/layer-9 [106-111]. Maps produced using the SAGA software (version 8.1.1).

***Journal_3: Figure 4: Unfortunately, the source for Figure 4 states that it has restricted use of attribution, this figure will need to be removed or replaced with a CC-BY 4.0 compliant source.***

CA: Ok, base map in Figure 4 has been replaced. The caption has been modified as follows: “Fig. 4. The three experimental designs employed in the present study: (a) ‘Upstream areas Exp_1’, (b) ‘Sources and sinks Exp_2’, (c) ‘Hazard areas Exp_3’. The coloured areas delimited in the three experimental designs were used to extract values from the feature variables. This methodology was replicated for each of the 73 sediment samples (Fig. 1b). Base Maps: Hillshade Map derived from DTM 5x5, Ed. 2014 (© Archivio Cartografico, Regione Emilia-Romagna; <https://metasfera.regione.emilia-romagna.it/ricerca_metadato?uuid=r_emiro:2016-08-08T155835>) and Technical Regional Map 1:5,000 (© Archivio Cartografico, Regione Emilia-Romagna; <https://metasfera.regione.emilia-romagna.it/ricerca_metadato?uuid=r_emiro:2017-11-24T113154>). Maps produced using the Esri© ArcMap software (version 10.3.1; ArcGIS)”.

Sincerely,

Manuel La Licata

(*Corresponding Author*)
